# Different coatings on magnetic nanoparticles dictate their degradation kinetics in vivo for 15 months after intravenous administration in mice

**DOI:** 10.1186/s12951-022-01747-5

**Published:** 2022-12-28

**Authors:** Yadileiny Portilla, Yilian Fernández-Afonso, Sonia Pérez-Yagüe, Vladimir Mulens-Arias, M. Puerto Morales, Lucía Gutiérrez, Domingo F. Barber

**Affiliations:** 1 Department of Immunology and Oncology and the NanoBiomedicine Initiative, Centro Nacional de Biotecnología (CNB)/CSIC, Darwin 3, Cantoblanco, 28049 Madrid, Spain; 2 Departamento de Química Analítica, Instituto de Nanociencia Y Materiales de Aragón (INMA), Universidad de Zaragoza, CSIC and CIBER-BBN, 50018 Zaragoza, Spain; 3 Department of Energy, Environment and Health, Instituto de Ciencia de Materiales de Madrid (ICMM-CSIC), Sor Juana Inés de La Cruz 3, 28049 Madrid, Spain; 4 Present Address: Integrative Biomedical Materials and Nanomedicine Laboratory, Department of Medicine and Life Sciences (MELIS), Pompeu Fabra University, Carrer Doctor Aiguader 88, 08003 Barcelona, Spain

**Keywords:** Iron oxide nanoparticle, Surface coating, Biodegradation, Biodistribution, Biotransformation

## Abstract

**Background:**

The surface coating of iron oxide magnetic nanoparticle (MNPs) drives their intracellular trafficking and degradation in endolysosomes, as well as dictating other cellular outcomes. As such, we assessed whether MNP coatings might influence their biodistribution, their accumulation in certain organs and their turnover therein, processes that must be understood in vivo to optimize the design of nanoformulations for specific therapeutic/diagnostic needs.

**Results:**

In this study, three different MNP coatings were analyzed, each conferring the identical 12 nm iron oxide cores with different physicochemical characteristics: 3-aminopropyl-triethoxysilane (APS), dextran (DEX), and dimercaptosuccinic acid (DMSA). When the biodistribution of these MNPs was analyzed in C57BL/6 mice, they all mainly accumulated in the spleen and liver one week after administration. The coating influenced the proportion of the MNPs in each organ, with more APS-MNPs accumulating in the spleen and more DMSA-MNPs accumulating in the liver, remaining there until they were fully degraded. The changes in the physicochemical properties of the MNPs (core size and magnetic properties) was also assessed during their intracellular degradation when internalized by two murine macrophage cell lines. The decrease in the size of the MNPs iron core was influenced by their coating and the organ in which they accumulated. Finally, MNP degradation was analyzed in the liver and spleen of C57BL/6 mice from 7 days to 15 months after the last intravenous MNP administration.

**Conclusions:**

The MNPs degraded at different rates depending on the organ and their coating, the former representing the feature that was fundamental in determining the time they persisted. In the liver, the rate of degradation was similar for all three coatings, and it was faster than in the spleen. This information regarding the influence of coatings on the in vivo degradation of MNPs will help to choose the best coating for each biomedical application depending on the specific clinical requirements.

**Graphical Abstract:**

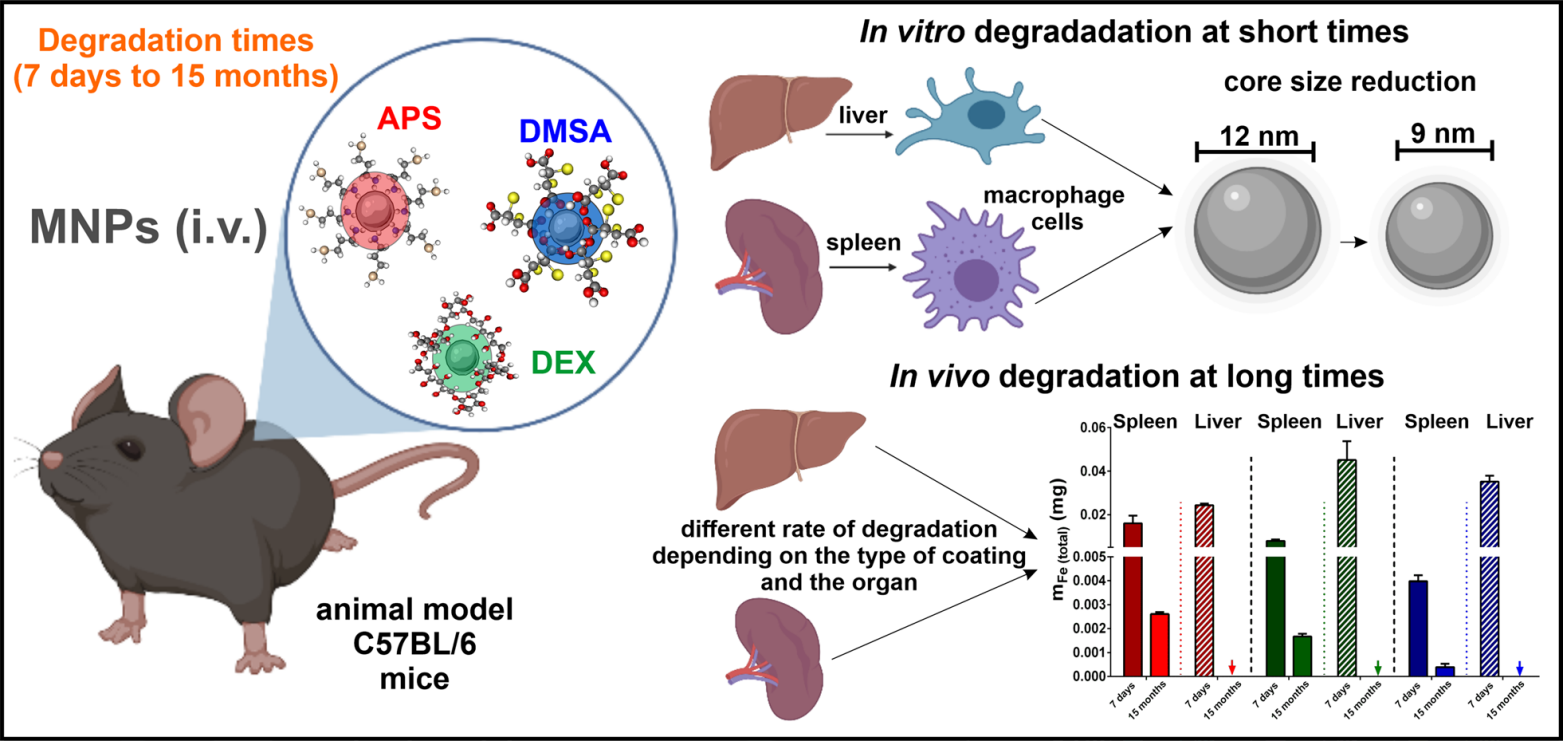

**Supplementary Information:**

The online version contains supplementary material available at 10.1186/s12951-022-01747-5.

## Background

Magnetic nanoparticles (MNPs) are one of the most widely used nanotechnological tools in biomedicine, mainly due to their nanometric size, optical, thermal and magnetic properties, and the fact that they can be manipulated with an external magnetic field [1, 2]. These characteristics favor their use in a variety of applications and accordingly, MNPs are useful as biosensors [3–5], as a sorbent in magnetic separation [6, 7], for in vivo diagnostic imaging [8, 9], in tissue repair [10] and in prostheses [11, 12]. In the ongoing battle against cancer, we can also highlight the therapeutic promise of MNP in hyperthermia [13–15] and for selective drug transport [16–19].

Iron oxide MNPs have been those of choice for a number of potential biomedical applications, given the feasibility of tracking them in tissues due to their magnetic response to an external magnetic field and their low toxicity [20]. Likewise, iron is easily metabolized by organisms, making iron oxide MNPs a viable candidate in minimally invasive methods for the diagnosis and treatment of diseases [21, 22]. Despite the success achieved using MNPs in animal models, to fully evaluate the clinical versatility of MNPs in biomedicine, it is important to understand both their short-term behavior in the body (blood circulation time [23, 24], biodistribution and degradation [25, 26]), as well as their biodegradability and toxicity in the long-term [27]. Using several MNP tracking techniques, such as transmission electronic microscopy (TEM), inductively coupled plasma optical emission spectroscopy (ICP-OES) and ferromagnetic resonance, after administration in vitro it has been seen that MNPs that enter cells tend to accumulate in lysosomes [28]. The magnetic properties of the particles inside the cell are affected over time, indicating they undergo at least some degree of degradation in the lysosomes [28–30]. In some cases, the biotransformation of MNPs into other iron species like ferritin was witnessed, suggesting that endogenous iron metabolism is involved in the biotransformation of MNPs [31–34].

To understand how MNPs are degraded in vivo, MNP biodistribution and the changes to their physicochemical properties at different time intervals after intravenous (i.v.) administration have been analyzed, mostly in mice [28, 29, 31, 32, 35, 36]. After i.v. administration, MNPs tend to accumulate mainly in the liver and spleen, where they are captured by macrophages of the mononuclear phagocytic system [29, 35]. Internalized MNPs are often seen to be degraded, although these studies have mostly been performed over short periods of time, days, weeks or a few months at best. Thus, the monitoring of MNP degradation is often incomplete as this would require monitoring the particles continuously until they completely disappear.

Comprehensive studies of MNP iron biotransformation in vivo are still very limited. Some of them reported the effect of a few properties of the MNPs on their degradation [31, 36–38], with their coating standing out as a possible key feature that regulates their biotransformation [36, 39]. A recent study comparing nanoparticles (NPs) of different sizes, coatings and internal architectures showed that smaller particles degrade faster. Moreover, particles of the same size degrade faster when they have acidic coatings relative to MNPs coated with other polymers [27]. However, given the diversity of the NPs available (shape, size, core composition, coatings) it is often difficult to compare between studies even if they were to follow the biotransformation process through to complete degradation as opposed to just focusing on relatively short time periods. In our lab, we previously analyzed how APS, DEX and DMSA coatings affect the formation, composition and degradation of the protein coronas (PCs) that form on MNPs in a biological milieu [40], and how APS and DMSA coatings influence their intracellular trafficking [30].

Here, we analyzed in vivo degradation of iron oxide nanoparticles with different coating after intravenous administration simulating an antitumor treatment. For these studies we used 12 nm iron oxide MNPs synthesized by the co-precipitation method and coated with three different molecules: 3-aminopropyl-triethoxysilane, APS-MNPs that have a positive surface charge; dextran, DEX-MNPs with an almost neutral surface charge; and dimercaptosuccinic acid, DMSA-MNPs that have a negative surface charge. First, we analyzed the biodistribution of these MNPs in vivo after their administration to C57BL/6 mice, using histology and magnetic susceptibility to test for their presence in the blood, spleen, liver, lungs, kidneys, heart, brain and thymus. Subsequently, we determined how the physicochemical properties of the MNPs (core size and magnetic properties) changed as they underwent intracellular degradation following internalization in two mouse cell lines: RAW 264.7, a murine circulating macrophage-like cell line; and NCTC1469, a mouse liver-derived macrophage-like cell line. After incubation of these cells with MNPs, endolysosomes loaded with MNPs were isolated and any changes to the iron oxide core size were assessed by TEM. From these studies we concluded that the decrease in size of the iron core differs depending on its coating, and there is also a correlation between the in vitro intralysosomal degradation of the MNPs and that which occurs in vivo in the macrophages of the organs where they are degraded. We then analyzed the in vivo degradation of these MNPs in C57BL/6 mice to which they were administered i.v. over 5 doses. The distribution of the MNPs and their magnetic properties in the organs where they accumulate were evaluated by dynamic magnetic measurements from one week and up to 15 months, a method proven to detect and quantify MNPs in tissues [41, 42]. These analyses showed that MNPs coatings play a key role on the short-term and long-term fate of MNPs in vivo, suggesting that the surface charge provided by the different molecules used to coat MNPs, together with the chemical nature of such molecules and the chemical bonds between such molecules and MNP surface or between such molecules and the biological milieu may influence on the MNPs biodistribution and degradation process.

Currently, few studies have followed the in vivo degradation of MNPs with different coatings for as long as 15 months after their administration [27]. Our studies provide clear evidence that the accumulation of MNPs in certain organs is dictated to a greater or lesser extent by the particle’s coating. As such, these studies could help determine the risks involved in the biomedical use of this type of MNPs, as well as defining the type of coating best suited to distinct therapeutic applications.

## Materials and methods

### Superparamagnetic iron oxide nanoparticles

*Physicochemical characterization of the MNPs.* Iron oxide core size and shape was determined by TEM. Images were captured on a 100 keV JEOL-JEM 1010 microscope equipped with a Gatan Orius 200 SC digital camera (Japan) and they were analyzed using ImageJ software (NIH, USA) to determine the MNPs' size, shape and distribution. After the coating process, a Zetasizer nano ZS (Malvern Instruments, Malvern, UK) was used to determine both the hydrodynamic size and Z-potential. The iron concentration was measured by ICP-OES (PerkinElmer) after acid digestion. For magnetic characterization, liquid samples were placed on a piece of cotton and allowed to dry at 50 ℃ overnight. The cotton samples were then placed inside a standard capsule for magnetic measurements and hysteresis loops with a maximum field of 5 T were measured in a Vibrating Sample Magnetometer (MLVSM9, MagLab 9 T, Oxford Instruments, UK). In addition, to prepare samples with different degree of dipolar interactions [43] the particles were diluted in hot agar (1% w/v) and allowed to cool down to RT in an ultrasound bath [44]. Solid agar solutions were then freeze-dried and placed into gelatin capsules for magnetic characterization using a QuantumDesign MPMS-XL SQUID magnetometer. AC magnetic susceptibility measurements were obtained with a field amplitude of 326 A/m and a frequency of 11 Hz in the temperature range from 2 to 350 K.

### Cell culture

The murine macrophage-like NCTC1469 (ATCC: CCL-9.1) and RAW 264.7 (ATCC: TIB-71) cell lines were cultured as described previously [29, 30]. These cells were maintained under standard culture conditions: 37 °C, 5% CO_2_, and 90% relative humidity.

### Study of in vitro MNP degradation in endolysosomal vesicles

*Isolation of MNP loaded endolysosomes.* The endolysosomal vesicles in which MNPs accumulate and are degraded were isolated following a previously described protocol [6, 30]. Briefly, cells were seeded at a density of ~ 7 × 10^6^ in a petri dish (P-100, Falcon) and both cell lines were then exposed to APS, DEX or DMSA coated MNPs at a concentration of 125 μg Fe/ml for 24 and 72 h at 37 °C. After recovering the cells by centrifugation (1200 rpm, 5 min at RT), they were resuspended in protein buffer (5 mM Tris base, 1 mM EDTA with a protease inhibitor cocktail: Roche) and the cell membrane was lysed mechanically by multiple passages through a G-22 needle attached to a 1 ml syringe. The percentage of cells with a broken cell membrane was evaluated by staining with Trypan Blue and optical microscopy, performing mechanical lysis until most of the cells (around 80–90%) were stained. To recover the endolysosomes containing the MNPs, the cell lysates were exposed to a magnet for 30 min at 4 °C and after centrifugation (1200 rpm, 5 min at 4 °C), the supernatant was discarded and the pellet was resuspended in 500 μl of the aforementioned protein buffer. The MNPs were resuspended in lysis buffer (1% Triton X-100, 1 mM EDTA with a protease inhibitor cocktail: Roche) and the total protein concentration in each extract was quantified using the BCA kit (ThermoFisher). The protein lysates obtained from the endolysosome-enriched fractions of the RAW 264.7 or NCTC1469 cells were analyzed in Western blots to verify this enrichment, probing the membranes with antibodies against the Lamp1 marker (Lysosomal associated membrane protein 1: SAB3500285, Sigma).

### Size of MNPs accumulated inside endolysosomes

The particle core size was determined from TEM micrographs acquired on a JEOL-1011 transmission electron microscope (100 kV). After being extracted from endolysosomes the particles were dispersed in water, and a drop of the suspension was placed onto a copper grid, covered by a carbon film and allowed to dry at RT. Microscopy images were taken by transmission electron TEM (a total of 30 images per type of MNPs and iron oxide core) of both the magnetic core and the MNPs once coated and the size of the magnetic core, their shape and distribution were analyzed using Image J software counting between 100 and 200 particles per image.

### Analysis of MNP magnetization before and after their internalization in endolysosomes

Liquid samples (100 μl) were placed on a piece of cotton and allowed to dry at 50 ℃ overnight. The cotton samples were then placed inside a standard capsule for magnetic measurements. Three different samples from three different experiments were measured for each condition (coating and cell line) and the magnetic curves were recorded between ± 5 T at 0.3 T/min at RT with a vibrating sample magnetometer.

### Mouse model

Female C57BL/6 mice (5-weeks-old: Envigo Laboratories) were maintained at the CNB animal facility and randomly divided into four groups of 7 animals, each receiving 5 intravenous injections of phosphate-buffered saline (PBS, control), APS, DEX or DMSA coated MNPs (25 mg Fe/kg/injection) over two weeks under 0.5–5% isoflurane inhaled anesthesia. At several time points after the last injection, the mice were sacrificed, and their spleen, liver, lungs, kidneys, thymus, heart, brain and blood were harvested for analysis. All animal studies were approved by the Ethics in Animal Experimentation Committee at the National Center for Biotechnology (CEEA-CNB), the Spanish Scientific Research Council (CSIC) Ethics Committee, and by the Division of Animal Protection of the Comunidad Autómoma de Madrid (CAM) in compliance with national and European Union legislation.

### Blood biochemical analysis

Blood samples were maintained at RT (~ 4 h), centrifuged (1500 rpm, 45 min at RT) and the serum was collected. The serum samples were analyzed for alanine aminotransferase and aspartate aminotransferase and other parameters indicative of liver toxicity at independent laboratories (SeroLab and Dynamimed). The blood samples were also analyzed by the Serolab and Dynamimed independent laboratories, focusing on the count of the leukocyte formula at each degradation time and for each treatment group in all cases.

### Histological Prussian blue staining to detect iron in paraffin tissue sections

After extraction, the organs were fixed in 4% paraformaldehyde (PFA), included in paraffin blocks, and sections (~ 7 μm) were then deparaffinized and rehydrated for staining (procedures carried out in collaboration with the CNB-CSIC Histology Service). Prussian blue staining was performed for approximately 15 min using equal volumes of 10% w/v HCl (Merck) and 10% w/v potassium ferrocyanide (Sigma), counterstaining with 0.01% w/v filtered neutral red (Sigma) for 2 min. The sections were washed in running tap water (10 min), dehydrated and mounted with Fluoromount-G (ThermoFisher). Images were acquired on an Olympus IX70 inverted brightfield microscope with 20 and 40X objectives.

### Immunohistochemistry of Kupffer cells of the liver

Immunohistochemistry was performed on murine liver tissue sections previously embedded in paraffin to assess whether MNPs accumulated and/or were degraded over time in liver macrophages. For this, the sections were first deparaffinized and rehydrated with xylene, then a series of ethanol solutions and finally, in distilled water (as described previously). The sections were then blocked for 1 h at 37 °C with 5% BSA (Sigma-Aldrich), before probing them overnight at 4 °C with the primary antibody F4/80 (14–4801-81, eBioscience). The following day, and after 3 washes with PBS, they were incubated with the secondary antibody for 45 min at RT and then for 30 min with a horseradish peroxidase (HRP)-streptavidin complex (KS001, Nanjing Jiancheng Bioengineering Institute) to detect the secondary antibody. Finally, the antibody staining was visualized with the AEC + solution for 15 min and iron staining was performed as described above. The samples were mounted with Fluoromont-G (SouthernBiotec) and examined under an Olympus IX70 inverted brightfield light microscope with 40 and 63X objectives.

### Qualitative and quantitative analysis of MNPs and ferritin in tissues using AC magnetic susceptibility measurements

Mouse tissue was freeze-dried overnight and all the organs (except the liver) were transferred directly to gelatin capsule sample holders for magnetic characterization. Given the large volume of the liver, this organ was ground in a mortar to obtain a homogenous powder and an aliquot (≈ 100 mg) of this powder was then placed inside a gelatin capsule for magnetic characterization. The temperature dependence of the AC magnetic susceptibility was measured using a QuantumDesign MPMS-XL SQUID magnetometer, using the AC option, a field amplitude of 326 A/m and a frequency of 11 Hz. Measurements were made over the temperature range of 2–350 K to identify and quantify the ferritin and MNPs present in the tissues. In addition, measurements were taken from other samples in the 200–250 K temperature range to locate the MNP maxima signal in out-of-phase magnetic susceptibility (χ″), or in the 2–40 K temperature range to locate ferritin.

The agar suspensions of the injected MNPs (see *MNP physicochemical characterization* section) and a mouse ferritin sample [45] were used as standards to quantify both species using the height of the AC susceptibility maxima, as described previously [42].

### Statistical analysis

All data are presented as the mean ± standard deviation (SD). All quantifications were performed in triplicate or in duplicate and the statistical analysis was performed with GraphPad Prism Software (CA, USA) and Origin 9.0, employing Kruskal–Wallis tests. The levels of significance are presented as: * p < 0.05, ** p < 0.01, and *** p < 0.001.

## Results and discussion

### Physicochemical characterization of iron oxide MNPs with different coatings

In this study we examined in mice the biodistribution and the biotransformation of MNPs with iron oxide cores of the same size but coated with APS, DEX or DMSA. The iron oxide MNPs were synthesized by co-precipitation method following the protocols described previously [46] and after core synthesis, a standard protocol was used to oxidize magnetite to maghemite activating the MNPs surface for coating [47, 48]. Although these types of MNPs have been used in previous studies of our group [30, 40, 49] we present here the physicochemical characteristics of the batches prepared for this new study, which may differ slightly from data obtained from other batches used in earlier works. In brief, TEM images revealed them to be monodisperse iron oxide MNPs ~ 12.0 ± 1.2 nm in diameter. The different coatings of these iron oxide cores produced MNPs with different surface charges, positive (APS), neutral (DEX) and negative (DMSA: Additional file 1, Fig. S1 a, b). In brief, TEM images revealed them to be monodisperse iron oxide MNPs ~ 12.0 ± 1.2 nm in diameter. The different coatings of these iron oxide cores produced MNPs with different surface charges, positive (APS), neutral (DEX) and negative (DMSA: Additional file 1: Fig. S1 a, b).

The hydrodynamic radius of the APS, DEX and DMSA coated-MNPs were 122 nm, 109 nm and 83 nm, respectively (Additional file 1: Fig. S1c) [40]. Thus, in addition to producing different surface charges, these coatings also affected the final size of the MNPs in suspension. The MNPs appeared to form small aggregates, although in all cases a single monomodal size distribution was recorded with polydispersity index (PDI) < 0.3 [48]. The Z-potentials confirmed the surface charge of the APS (+ 23 mV), DEX (-1.8 mV) and DMSA (-34 mV) coated MNPs (Additional file 1: Fig. S1d) [40]. Moreover, the M(H) hysteresis loop confirmed their superparamagnetic behavior at room temperature (RT). Finally, the saturation magnetization values of all the samples were ~ 80 Am^2^/kg_Fe_ (Additional file 1: Fig. S1e), consistent with previously reported values for γ-Fe_2_O_3_ nanoparticles [40] and in agreement with the chemical structure determined by Mössbauer spectroscopy of particles prepared by this methodology [50, 51].

The AC magnetic susceptibility measurements were temperature dependent (50–350 K range) and the typical relaxation phenomenon of MNPs was observed, an in-phase magnetic susceptibility maxima [χ’ (T)max] together with an out-of-phase magnetic susceptibility [χ″(T)] maximum at slightly lower temperatures (Additional file 1: Fig. S1f). The maximum of the MNPs with each of the coatings was detected at slightly different temperatures depending on the interparticle dipolar interactions and in agreement with the dynamic light scattering (DLS) results, being the DMSA coated particles the ones presenting the lower degree of aggregation. The temperature of these maxima (190–220 K range) was used as a fingerprint of the presence of the MNPs in animal tissues. Furthermore, the susceptibility per mass of iron in the form of particles was used to quantify the MNPs in tissues [43].

### Experimental study of iron oxide MNP degradation in vivo

**Fig. 1:**
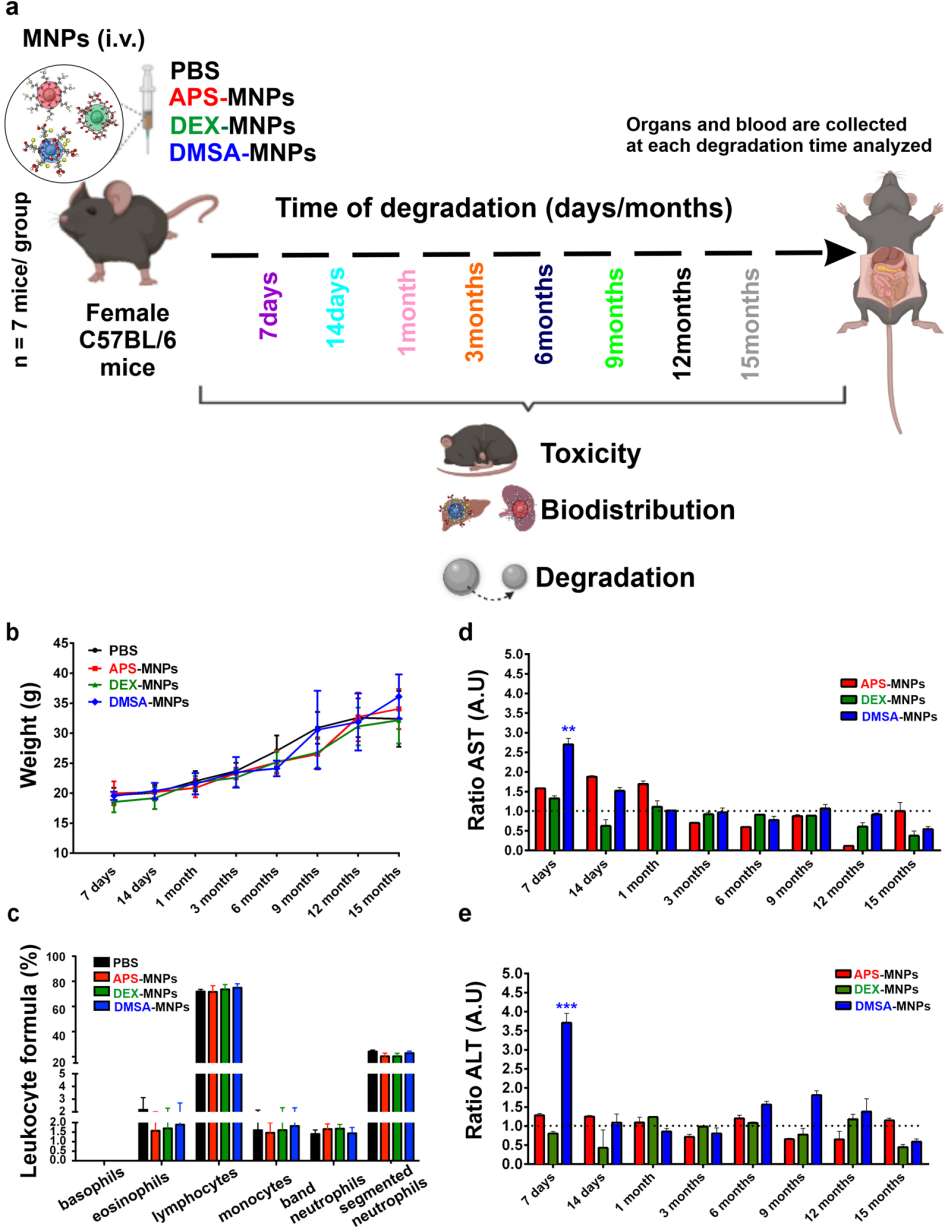
In vivo toxicity of iron oxide MNPs coated with different molecules. **a** Scheme of the experimental design of the in vivo study of MNP toxicity and degradation. **b** Body weight of the mice treated with PBS (control) or the APS, DEX or DMSA coated MNPs monitored over a 15-month period after treatment. **c** Leukocytes in the C57BL/6 mice treated with PBS and the different MNPs. **d**,**e** Hepatic profile (aspartate aminotransferase -AST), alanine aminotransferase -ALT) in blood samples collected at different times post-administration from 7 mice each treated with PBS (control) or the APS, DEX or DMSA coated MNPs. The hepatic data were normalized to that of the control group administered PBS and the data are shown as the mean ± SD (n = 7) of each group at each time point. The dashed dotted lines show the normalization of the values with respect to the control (group of mice administered PBS). Kruskal–Wallis test: *p < 0.05, ** p < 0.01 and *** p < 0.001

MNP degradation was studied in vivo in 5-week-old female C57BL/6 mice (Envigo Laboratories) maintained under controlled conditions at the National Center for Biotechnology (CNB) animal facility. The mice were randomly divided into four groups of 7 animals, that each received five doses (at a twice weekly frequency) of PBS (control), APS, DEX or DMSA coated MNPs (100 μl of MNPs, 2.5 mg Fe/mice) by retro-orbital i.v. injection under isoflurane anesthesia (0.5–5% inhaled). This dose schedule is the same schedule that we routinely use for MNP injection to treat tumors in mouse models of cancer [17, 52]. After administration of the MNPs, different health parameters were evaluated in all the mice, including weight, physical appearance, blood cell populations and hepatic toxicity profile. In all cases, these results were compared to the control group that received PBS alone (Fig. 1a). To evaluate the possible toxic effects of the APS, DEX or DMSA coated MNPs, the appearance of several signs of systemic toxicity was evaluated over the 15-month study period, assessing bradykinesia or lethargy, piloerection, gastrointestinal symptoms and irregular breathing. No signs of acute toxicity were observed at any of the time points analyzed after the administration of the different coated MNPs.

The evolution of the body weight of the mice over the entire experiment showed no significant differences among the groups (Fig. 1b) and the leukocyte profile was very similar between the mice treated with MNPs and the untreated mice, indicating there were no infections or symptoms of toxicity in mice inoculated with the different coated MNPs (Fig. 1c). The serum from mice injected with APS-, DEX- or DMSA-MNPs showed transient increases in aspartate aminotransferase (AST) and alanine aminotransferase (ALT) relative to the controls, enzymes associated mainly with hepatic damage. The increase in AST peaked from 7 days to 1-month post-treatment (Fig. 1d), whereas the increase in ALT was only produced by DMSA-MNPs at 7 days (Fig. 1e). However, these increases did not compromise the survival of the mice and the levels detected were within the range of normal values, similar to those detected in the control female C57BL/6 mice considering their age and the blood extraction method (~ 213.57 ± 38.42 U/L).

From the results obtained, we concluded that none of the MNPs were toxic to the mice over the observation period at the doses used. After sacrificing the animals, the size, appearance and color of the internal organs was apparently normal in all cases. Indeed, similar results were observed previously where DMSA-coated NPs synthesized by decomposition in an organic medium or DMSA-MNPs obtained by co-precipitation were administered to mice, although some mild toxicity was observed at 7 days in these earlier experiments that normalized over time, supporting our conclusion that this type of MNP did not cause toxicity over a period of 90 days [29, 53].

### Biodistribution of the iron oxide MNPs with different coatings in mouse tissues

To determine whether the NP coating influenced the biodistribution of the MNPs, we assessed whether the MNPs were still circulating in the blood or if they were located in the organs, and where they tended to accumulate 7 days after the last dose administered. Thus, the amount of iron in the blood was determined by ICP-OES, comparing each of the coated MNPs with the controls. The iron detected in the blood of the mice treated with the MNPs was lower than that detected in the untreated control mice (Additional file 1: Fig. S2), which would appear to reflect the regulation of iron metabolism after MNP internalization [54, 55].We previously found that in magnetic susceptibility analyses DMSA-coated NPs were not detected in the blood between 30 min of administration and up to 90 days [29]. Elsewhere the half-life of NPs with similar coatings in the blood was reported to be between minutes and 62 h depending on the coating and animal model used [56]. Hence, the blood residence time of the MNPs appears to be less than 7 days irrespective of the coating they carry. In fact, 7 days after administration the MNPs had accumulated in the different organs in which they were distributed [36, 57, 58], which led us to analyze the biodistribution of all the MNPs at this time.

**Fig. 2:**
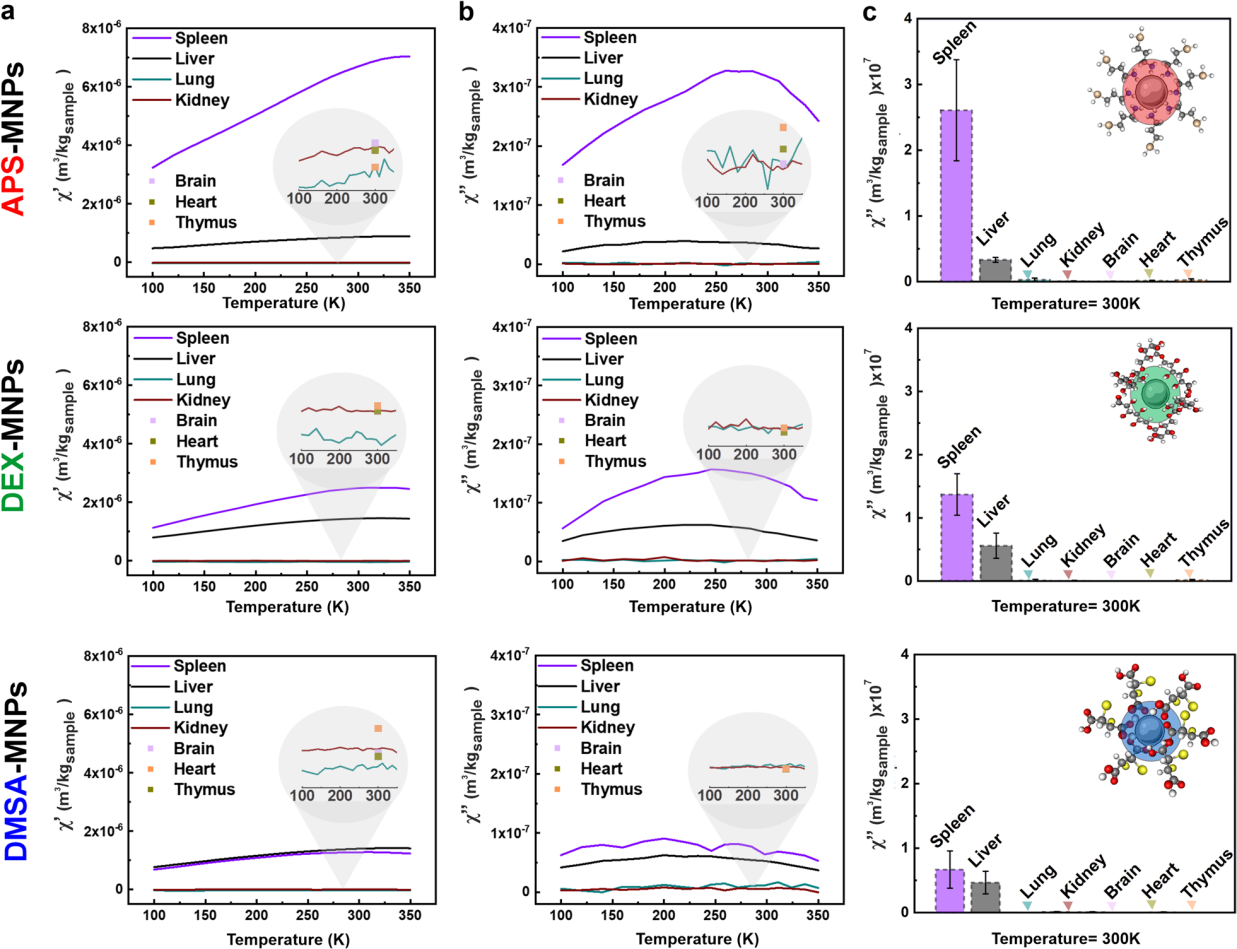
Biodistribution of MNPs with different coatings 7 days after the last dose administered to C57BL/6 mice. **a**, **b** Temperature dependence of the AC magnetic susceptibility: **a** in-phase and **b** out-of-phase components of murine tissues from mice treated with MNPs with the different coatings at 7 days post-administration. The height of the maximum of these signals is a surrogate indicator of the concentration of the particles in the tissues. The different organs are represented as continuous colored lines: spleen, purple; liver, black; lung, blue; and kidney, red. A single point was measured at room temperature for the brain, heart and thymus, as indicated in pink, green and orange, respectively. **c** The height of the out-of-phase magnetic susceptibility maximum at 300 K in spleen, liver, lung, kidney, brain, heart and thymus. Of the total number of mice used for each treatment group (n = 7), 4 mice per group were analyzed and the data obtained is shown as the mean ± SD (n = 4)

The biodistribution of APS, DEX or DMSA coated MNPs was studied in C57BL/6 mice using AC magnetic susceptibility measurements after i.v. administration of five doses. This technique was especially relevant as the susceptibility maxima serves as a fingerprint of the presence of particles in a given tissue [42] and it has not only been used previously for biodistribution studies but also, to follow the biotransformation of MNPs over time [17, 29, 35, 39]. In our experiments, spleen, liver, kidney, lung, heart and thymus tissues were characterized magnetically to track MNP accumulation. The AC susceptibility signal obtained from the MNPs in the spleen and liver was detected at similar temperatures as when these MNPs were assessed in agar (compare Additional file 1: Fig. S1f with Fig. 2a, b), confirming the presence of the material administered in these tissues. We found minimal or no signal from the MNPs in lung, kidneys, brain, heart and thymus tissues, at least not within the limits of detection of the technique (~ 0.7 μg_Fe_: Fig. 2c). As expected, no signal from the particles was found in tissues from the control (PBS-treated) mice (Additional file 1: Fig. S3).

The initial biodistribution of MNPs depends on several factors, including their half-life in blood, the mouse strain, the injection dose, repetitive administration or the induction of anesthesia [29, 59, 60]. However, the physicochemical properties of the MNPs (surface charge, coating and the size of the core) exerts the greatest influence on the time of circulation in the blood [61]. Generally, iron oxide MNPs that exhibit long blood half-lives have limited distribution into the liver cells with significant uptake into the macrophage cells of other organs like the spleen, lymph nodes and bone marrow [62]. We found that 7 days after administration all the MNPs were localized in the spleen and liver, although a correlation was observed between the MNP coating and their final organ localization. Cationic surface APS-MNPs accumulated more in the spleen than in the liver, whereas anionic surface DMSA-MNPs and neutral DEX-MNPs accumulated similarly in the liver and spleen. Previously, it was proposed that stronger uptake of MNPs by liver macrophage and endothelial cells is related to a shorter circulation time of the particles in the blood [56, 63].

Positively charged particles like APS-MNPs may have longer circulation times in the blood than negatively charged particles [29, 56], which could explain the differences in the accumulation of APS and DMSA coated MNPs in the liver. In addition to the influence of the surface charge of the particle, its size is also an important factor to consider as smaller particles tend to remain in the bloodstream for longer [27]. By contrast, larger MNPs (> 50 nm in diameter) were more easily sequestered by macrophages in the liver and spleen [26, 64]. The MNPs used in this study had a hydrodynamic size between 165 and 1554 nm when incubated for 24 h with mouse serum (Additional file 1: Fig. S4), such that it was expected they would mainly be sequestered by macrophages in the liver and spleen, accumulating more strongly in these organs. Another issue to be considered is the composition of the PC as there are proteins like albumin and apolipoproteins that have a stabilizing effect, while others like fibrinogen trigger particle aggregation [40, 65]. Consequently, macrophages modified the internalization rate, the endocytic pathways used and the MNP uptake times [38, 66] based on the factors to which they have been previously exposed.

**Fig. 3:**
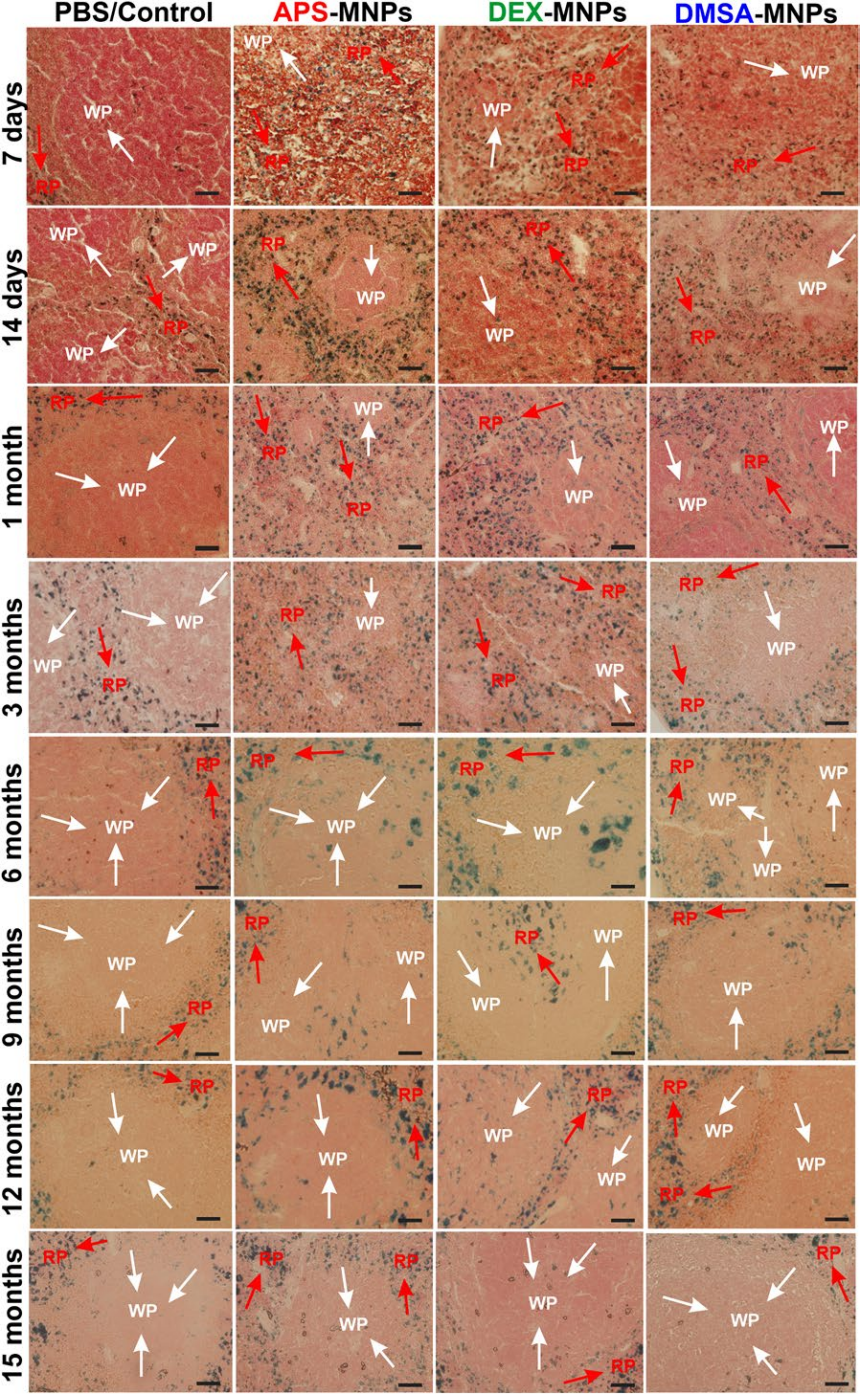
Prussian blue staining of spleen tissue sections from mice at different times after PBS (control), APS-, DEX- or DMSA-MNP administration. The areas of white pulp (WP) are indicated with white arrows and the red pulp (RP) with red arrows. Representative images of 10 tissue sections per condition. Scale bar 40 μm (20X) and zoom 40X. In all cases, it is compared using the control to which MNPs were not administered as a reference, inferring an increase in the presence of iron (marked in blue) as the presence of iron from the MNPs in the groups that were treated with the different types of MNPs. The data are shown as the mean ± SD (n = 7)

To complement the MNP biodistribution studies performed by AC magnetic susceptibility, mouse liver and spleen samples from treated C57BL/6 mice were stained using the Prussian blue technique 7 days to 15 months after i.v. MNP administration, showing iron accumulation in areas of both tissues by light microscopy (the iron that corresponds to the presence of MNPs was observed in blue, while cells were observed by counterstaining with neutral red). Prussian blue staining of spleen tissue sections showed iron accumulated mainly in the red pulp of the spleen following administration of any of the MNPs studied. Stained areas in the red pulp were evident in spleen sections, even in control tissues (Fig. 3), possibly due to the storage of iron degradation products as a result of erythrocyte phagocytosis and the presence of splenic macrophages [29, 67]. This accumulation of MNPs in the red pulp was expected as it is the spleen area that specializes in filtering the blood, eliminating old erythrocytes, pathogens or foreign elements. Many of the elements that circulate in the blood, such as aged erythrocytes, pathogens and MNPs, arrive transported by the arterial blood into the reticular fiber network of the spleen red pulp, where they are first retained and later phagocytosed by the many macrophages that are located at this reticular fiber network [67–69]. Since the spleen red pulp is a physiological storage site for iron, erythrocytes and platelets, iron stain was even observed in the red pulp of control mice [24]. The Prussian blue staining observed at 7 days in the spleen sections reflects this conclusion, along with the magnetic susceptibility measurements (Fig. 2). MNPs accumulate in the spleen in greater proportions when they were coated with APS as opposed to DEX and lastly, DMSA (Fig. 2).

From 7 days to 6 months after APS-MNP administration, iron was observed in both the red and white spleen pulp, and subsequently, a large amount of iron was internalized into the spleen until 9 months when it began to decrease. However, the iron signal did not disappear in the spleen of mice that received APS-MNPs. There was a gradual decrease of iron staining in the white pulp of spleen sections from mice that received DEX-MNPs after 6 months, and iron was observed for up to 3 months in both the red and white pulp of the spleen in mice that received the DMSA-MNPs, after which the amount of iron began to decrease. This appearance of an iron signal in the white pulp could be related to an excessively strong increase in iron in the spleen, which was more accentuated after the administration of APS-MNPs followed by DEX- and DMSA-MNPs. Hence, the iron signal persists longer in the white pulp of spleens in mice treated with APS-MNPs. Data from mice that received polyacrylic acid-coated NPs (PAA-NPs) was consistent with our results in which iron accumulated in both the white and red splenic pulp. No iron accumulated in the white pulp of the control mice, only in the red pulp, which might reflect the storage of iron degradation products as a result of erythrocyte phagocytosis [70].

**Fig. 4:**
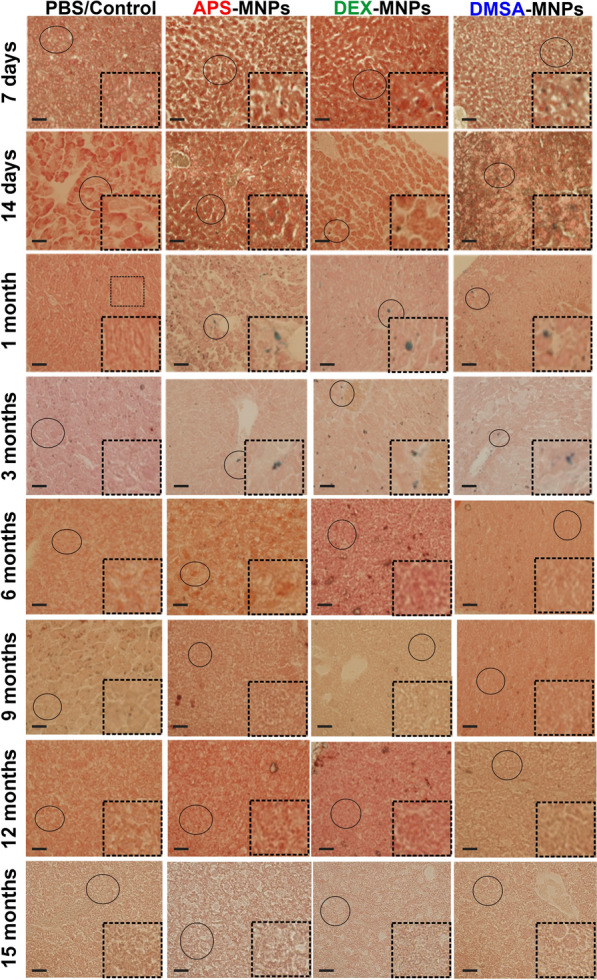
Prussian blue staining of liver tissue from mice treated with PBS (control), APS-, DEX- or DMSA-MNPs at different times post-administration. Representative images of 10 tissue sections per condition. Scale bar 40 μm (20X) and zoom 40X (bottom right inset). In all cases, it is compared using the control group, to which no MNPs were administered. In the control group no presence of iron is observed (marked in blue), iron is only observed in the groups to which MNPs were administered. The data are shown as the mean ± SD (n = 7)

In liver sections there was a reduction in the number of iron clumps (Fig. 4), as well as an increase in the size of the iron deposits over time, which might be explained by the formation of phagocytic cell clusters in the liver parenchyma [24]. In liver sections stained with Prussian blue iron complexes, a specific homogeneous distribution of iron throughout the liver sections was evident after short times (7 days or 1 month of MNP administration), which shifted to an accumulation close to the blood or bile ducts in mice treated with MNPs from 1 to 3 months. At longer times, between 6 and 15 months, the amount of iron observed in the liver decreased in all the sections analyzed (Fig. 4, bottom right insets). Apart from the accumulation of iron in the spleen and liver, we found no structural or histopathological changes in any of these tissues.

In summary, these results suggested that the type of coating plays a crucial role in the biodistribution of the MNPs, probably due to the changes induced in their physicochemical properties: surface charge, state of aggregation, hydrodynamic size and interaction with biological media [71–73]. In most cases, these differences dictate the amount of MNPs that accumulate in the different organs.

### Short term intracellular degradation of iron oxide MNPs with different coatings in endolysosomal vesicles

Although MNPs accumulated in the liver and spleen, regardless of their coating, their proportions in these organs and their degradation over time did seem to be influenced by the coating. Hence, the intracellular degradation of MNPs was studied within 24 h after internalization in two different macrophage lines: RAW 264.7 cells [30], a murine circulating macrophage-like cell line; and NCTC1469 cells, a mouse liver-derived macrophage-like cell line [29, 35]. Biodegradation studies identified a loss of the magnetic properties of MNPs after administration, which is correlated with an increase in iron metabolism suggesting their active degradation [74, 75]. The availability of iron derived from MNPs depends on the mechanisms by which nanoparticles are internalized by cells and how this internalization influences their degradation [35]. The highly proteolytic properties of endolysosomes, such as low pH, high ionic strength and the presence of various catabolic enzymes are primarily responsible for the degradation of nanomaterials [76, 77]. MNP degradation first occurs at the level of the PC associated with the MNP surface after coming into contact with blood or other biological fluids [40]. The coating or functionalization of the MNPs then degrades and finally, the metallic core disintegrates [78]. Each of these processes is influenced by the nature of the MNPs, the type of cell in which degradation occurs and the cell’s metabolic state [79].

**Fig. 5:**
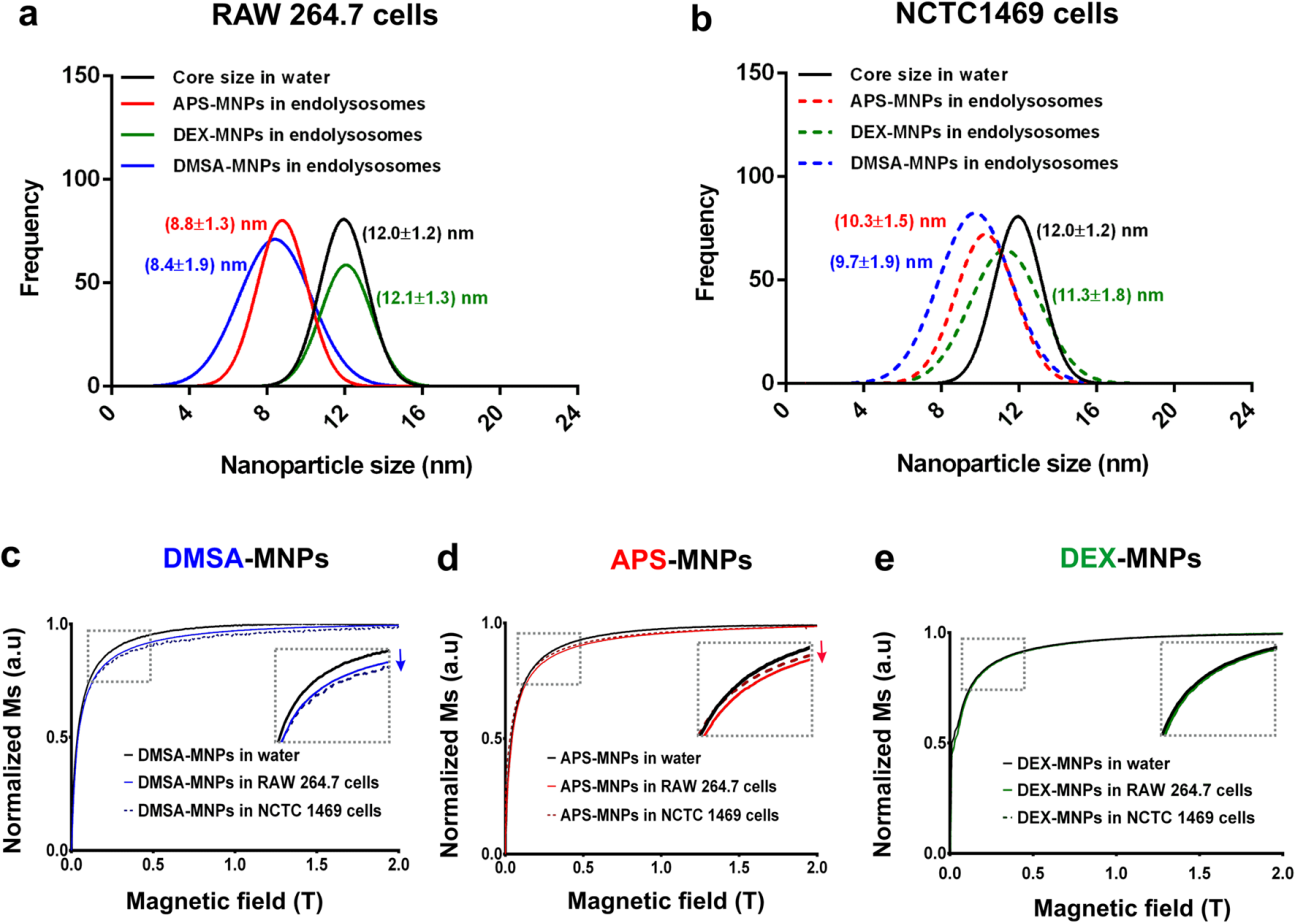
MNP degradation after endolysosome internalization in RAW 264.7 and NCTC1469 cells. Iron oxide core size for APS, DEX and DMSA coated MNPs in RAW 264.7 (**a**) and NCTC1469 cells (**b**). The core sizes were analyzed in 30 TEM images with Image J software. Magnetization of DMSA-MNPs (blue line, **c**) APS-MNPs (red line, **d**), DEX- MNPs (green line, **e**) and in water (black lines), within isolated endolysosomes in RAW 264.7 (continuous lines) and NCTC1469 cells (discontinuous lines) after a 24 h incubation

To assess whether the MNPs undergo different rates of intracellular degradation, we monitored the size of the cells magnetic MNP core within the endolysosomes 24 h after RAW 264.7 and NCTC1469 were exposed to these particles. Fractions were isolated that were enriched in the expression of *bona fide* endolysosomal markers and hence, of these organelles (Additional file 1: Fig. S5). Nevertheless, cell viability was not affected by MNP treatment even after a 24 h incubation with MNP iron concentrations up to 125 μgFe/ ml (Additional file 1: Fig. S6). To follow the core size reduction as an indication of MNP degradation by TEM, the mean particle size of over 100 MNPs was measured at 24 h (Additional file 1: Fig. S7). Considering the core size of the MNPs in water as the baseline (12.0 ± 1.2 nm: Fig. 5), there were differences in the intralysosomal degradation of the APS-MNPs between cell types, with a greater reduction of the core size in RAW 264.7 cells (8.8 ± 1.3 nm) than in NCTC1469 cells (10.3 ± 1.5 nm). DMSA-MNPs were rapidly degraded in both cell types, with a higher degradation rate than APS-MNPs in the liver-derived macrophage NCTC1469 line (9.7 ± 1.9 nm: Fig. 5 a, b). Finally, poor in vitro degradation of DEX-MNPs was evident in both cell types, perhaps related to their weaker internalization in these cells (Additional file 1: Fig. S8).

The influence on the reduction in the core size of each MNP was corroborated by field-dependent magnetization M(H) measurements. At RT, the M(H) curves of the MNP loaded endolysosomes displayed slight differences between cell types and MNP coatings (see Fig. 5). Common features of the endolysosome loaded MNP M(H) hysteresis curves relative to the MNP suspensions included a paramagnetic contribution over time (i.e.: a lineal increase in magnetization in the high magnetic field region), which was probably related to the presence of Fe ions, a decrease in saturation magnetization of the endolysosomes from the cells exposed to MNPs for 24 h and a reduction in magnetic susceptibility (dM/dH slope at low magnetic field) as a consequence of the reduction in MNP size. In general, there was more degradation of the DMSA-MNPs in both cell types, reflected in the reduced size of the core detected in TEM images and by the magnetization analyses (Fig. 5c). APS-MNPs were degraded more severely in the RAW 264.7 cells than in the hepatic NCTC1469 cells, indicative of greater degradation in spleen macrophages (Fig. 5d). Finally, no significant degradation of DEX-MNPs was observed in either of the two cell types analyzed (Fig. 5e), which could be related to the poor internalization of these particles given their almost neutral surface charge (Additional file 1: Fig. S1d).

It should be noted that 24 h is a short period to draw meaningful conclusions about the degradation of each MNP. However, it was impossible to monitor degradation beyond 24 h due to the multiplication of the cells in vitro and thus, we were only able to analyze this 24 h period of intralysosomal degradation. To study the disappearance of MNPs after their administration and the differences in degradation kinetics, degradation experiments in vivo should be performed over longer time periods, establishing the differences between coatings at the organ level.

### Degradation of iron oxide MNPs in the liver

To get a deeper understanding of the intracellular degradation of MNPs with different coatings in hepatic macrophage cells they were studied at longer times after administration to C57BL/6 mice. This analysis focused on liver macrophages as they showed greater differences in MNP degradation over short periods and approximately 30–99% of the MNPs administered accumulate in the liver after administration [80, 81]. To perform this analysis, specific F4/80 staining was studied in liver tissue sections between 7 days and 15 months from the administration of the last dose of the MNPs. After counterstaining the sections with Prussian blue and studying the co-localization of F4/80 with the iron signal, any decrease in this co-localization in hepatic macrophage cells was considered a sign of degradation.

The liver is a complex network of interrelated cells, with specialized epithelial cells, hepatocytes, representing approximately 60–80% of its parenchymal cells. Other liver cells include: Kupffer cells (KCs) and mobile macrophages, hepatic sinusoidal endothelial cells, hepatic stellate cells, biliary epithelial cells (cholangiocytes), resident immune cells (dendritic cells, natural killer cells, and lymphocytes) and circulating blood cells in transit through the liver. KCs represent 80–90% of the total body macrophage population and they are responsible for most of the phagocytic activity in the liver [82–84].

**Fig. 6:**
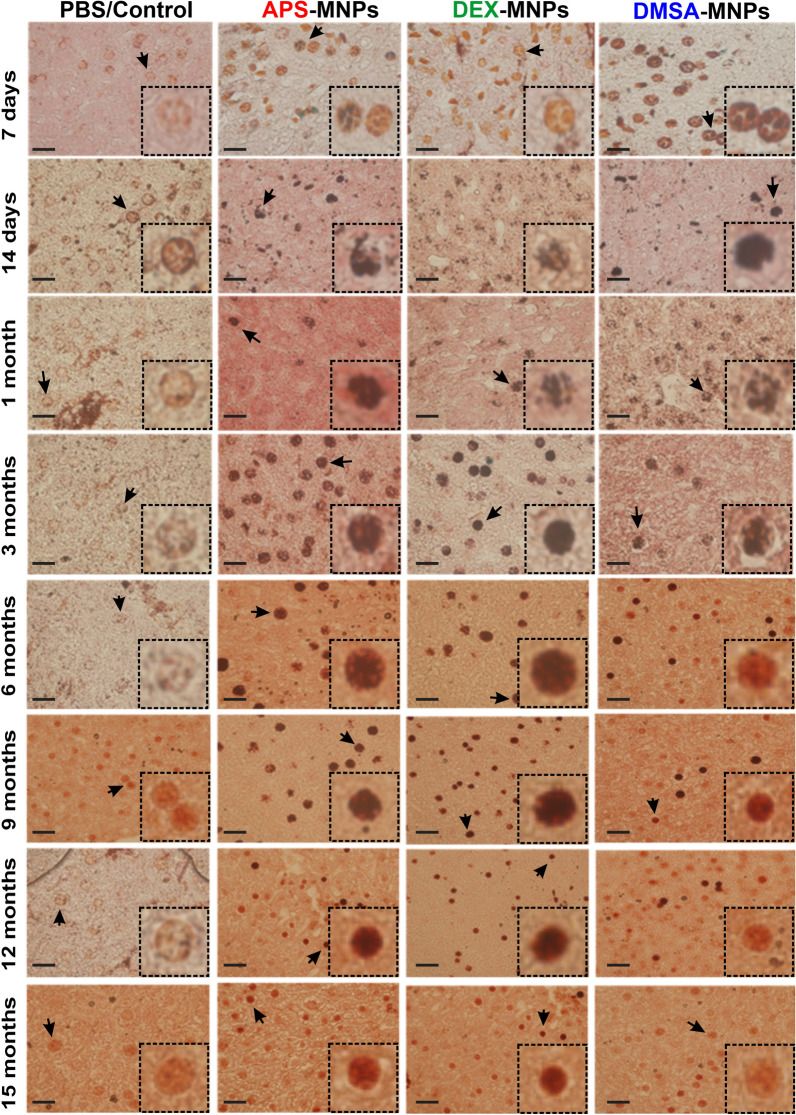
Degradation of MNPs in Kupffer cells from 7 days to 15 months post-administration of the MNPs with different coatings. Immunohistochemical analysis of MNP accumulation in Kupffer liver cells (labeled with the macrophage F4/80 antibody) and intracellular iron visualized by Prussian blue staining and a neutral red counterstain. In the lower right corner of each panel (dashed box) an amplified image of the Kupffer cells is shown. Representative images of 15 sections per condition are shown (n = 7). Scale bar 40 μm (20X), 63X or 100X (bottom right inset) zoom objective. In the images, the area where the zoom shown in the bottom right inset has been made has been marked with a black arrow. In the figures that the area in question is not indicated, it is because it coincides with the bottom right inset area where the image is placed or the zoom corresponds to another of the images

Using immunohistochemistry with F4/80 markers on paraffin embedded sections, we assessed whether the MNPs were located fundamentally in the liver macrophages or KCs, and where they were degraded [36, 61, 85]. Mainly DMSA and APS coated MNPs were partitioned early into KCs in the liver (see Fig. 6). When Prussian Blue staining/iron co-localization with the KC F4/80 macrophage immunolabeling was studied, a decrease in the iron staining in KCs was observed over time. Greater iron staining in KCs was observed at short degradation times for DMSA-MNPs, between 7 days (42.22%) and 1 month (77.78%). However, the increase in the presence of iron in KCs was not observed until 14 days with APS coated MNPs (55.55%) and 6 months for DEX coated MNPs (62.50%). When DMSA-MNP intracellular degradation was observed, 1 month after administration, there were fewer KCs in which iron was detected, whereas this decrease in KCs containing iron was not observed until 6 and 12 months for APS- and DEX-MNPs, respectively. These data suggested that DMSA-MNPs were degraded more rapidly in the liver than APS or DEX coated MNPs. Finally, when degradation was analyzed at the final time point (15 months), iron detected in KCs administered DMSA-MNPs (10.52% of the KCs containing iron) was similar to the iron detected KCs administered PBS (9.47% of the KCs containing iron). In the case of APS and DEX coated MNPs, the blue iron signal still co-localized with hepatic macrophage labeling after 15 months (21.82% for APS-MNPs and 38.46% for DEX-MNPs), suggesting a slower degradation of these MNPs.

The importance of KCs in innate immunity and the degradation of intracellular iron has been highlighted [61, 86]. KCs are liver resident macrophages that engulf and destroy pathogens, as well as other foreign bodies and materials in the blood. These macrophages are also involved in erythrocyte recycling and apoptotic cell digestion [80], and they are thought to recognize MNPs as foreign material and internalize them through multiple receptors [37]. The uptake and retention of MNPs by KCs is strongly correlated with their surface charge, the nature of their chemical coating and their size [87]. Larger particles are usually phagocytosed more easily by this cell type, while MNPs with strongly cationic and anionic surface charges adsorb a quantity of serum proteins to form their PC and can aggregate, interacting more readily with macrophages in vitro. Most surface-neutral ligands adsorb less serum proteins to their surface and they are therefore less efficiently absorbed by phagocytic cells than more charged nanoparticles [88] (Additional file 1: Fig. S4).

The differences in the MNP coating and the associated differences in the composition of the PC may explain why DEX-MNPs reside longer in KCs, as these MNPs were phagocytosed more slowly by macrophages due to their neutral charge [89, 90]. Regarding the influence of PC on the internalization of MNPs with different coatings in liver macrophages, we previously observed that the PC associated with APS and DMSA coated MNPs was more diverse in terms of size and composition, with the presence of complement proteins and immunoglobulins that favor the opsonization of MNPs by macrophages [91]. These results suggested that not only do MNPs accumulate in a higher proportion in the liver or spleen, depending principally on their coating and influenced by the hydrodynamic size of the MNPs, but also, that the MNPs seem to have different degradation rates in the liver at least, with anionic DMSA-MNPs being degraded faster than the cationic APS-MNPs.

### Long-term degradation of iron oxide MNPs with different coatings

**Fig. 7:**
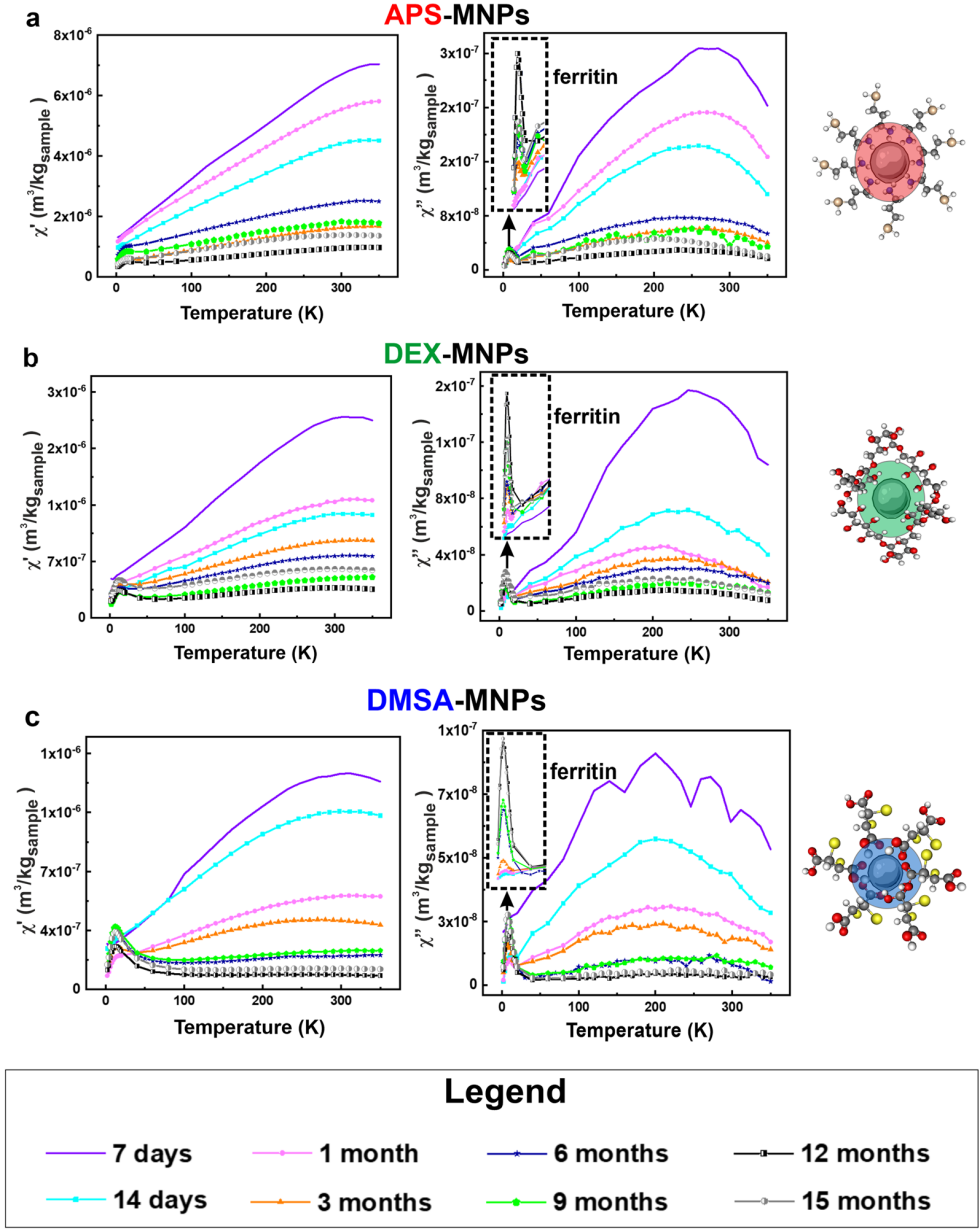
Evolution of the degradation of the APS, DEX and DMSA coated MNPs in the spleen as measured at different times by the AC magnetic susceptibility. The magnetic susceptibility profiles of the spleen extracted from the mice to which the different MNPs were administered: **a**, APS-MNPS; **b**, DEX-MNPs; and **c**, DMSA-MNPs. The signal corresponding to ferritin is indicated in the degradation profile of the different MNPs (upper left corner of the χ′′ profile). The different degradation times after the last dose administered are represented in the following colors: 7 days (purple), 14 days (cyan), 1 month (pink), 3 months (orange), 6 months (blue), 9 months (green), 12 months (black) and finally 15 months (gray). The in-phase (real, χ′- on the left) and out-of-phase (imaginary, χ′′- on the right) components of the AC magnetic susceptibility measurements are shown

**Fig. 8:**
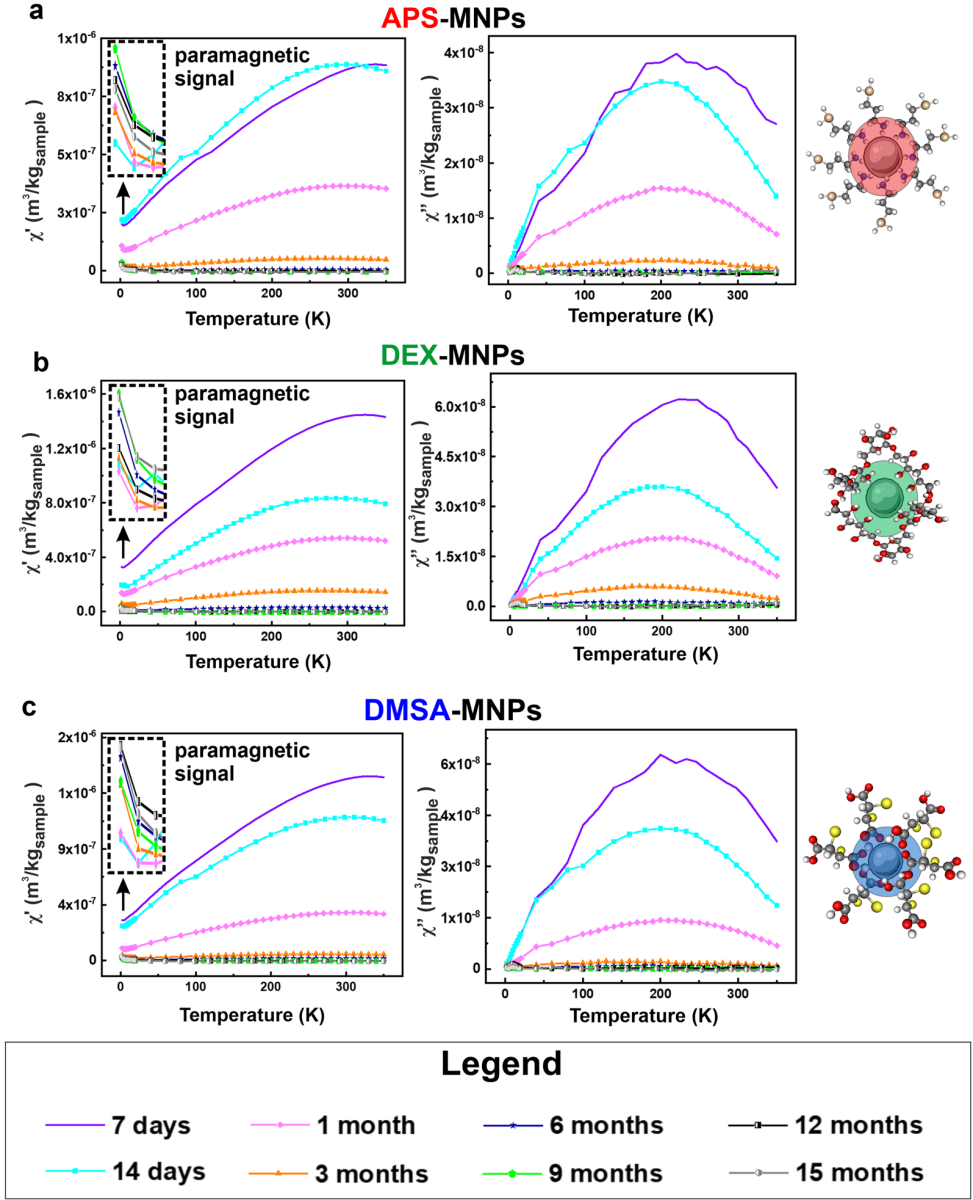
Evolution of the degradation of the APS, DEX and DMSA coated MNPs in the liver measured at different times by AC magnetic susceptibility. The magnetic susceptibility profiles of the liver in mice to which the different MNPs were administered: **a**, APS-MNPs; **b**, DEX-MNPs; and **c**, DMSA-MNPs. The different degradation times after the last dose administered are represented in the following colors: 7 days (purple), 14 days (cyan), 1 month (pink), 3 months (orange), 6 months (blue), 9 months (green), 12 months (black) and finally, 15 months (gray). The in-phase (real, χ′- on the left) and out-of-phase (imaginary, χ′′- **o**n the right) components of the AC magnetic susceptibility measurements are shown. The inset in the upper left corner of the χ′ profile shows the paramagnetic contribution observed at very low temperatures

In light of the above, we evaluated MNP degradation over 15 months by monitoring the magnetic susceptibility measurements in both the spleen and liver, the tissues in which the MNPs mainly accumulated. The long-term changes to the MNPs accumulated in these tissues was assessed by AC susceptibility from 7 days to 15 months post-administration. In general, MNPs were detected in the spleen tissues throughout the post-administration period analyzed, whereas MNPs were only detected in liver samples until 3- or 6-months post-administration depending on the NP coating (Figs. 7, 8). These results were consistent with the Prussian Blue staining data obtained for both these organs.

In addition, a new contribution from a different iron-containing species was detected 14 days after MNP administration in the spleen tissue, with a maximum in the out-of-phase susceptibility located at 8–10 K, accompanied by a maximum at slightly higher temperatures in the in-phase susceptibility. This signal was also observed in some of the hepatic tissue samples at longer time points and it corresponded to the typical signal of ferritin, the iron storage protein that enables iron to accumulate in a biomineral form inside the protein cage [43, 45, 75]. Moreover, a paramagnetic signal was observed in the in-phase magnetic susceptibility at low temperature in some samples of liver tissue. This may be attributed to other iron-containing species in which this element is not part of any mineral or biomineral form.

In this type of measurement, the height of the magnetic susceptibility maxima when plotted per tissue mass is directly related to the iron concentration in the tissue (m_Fe_/m_sample_). A decrease in the height of the susceptibility maximum implies a decrease in the number of particles in the tissue and/or their degradation. Here, the general trend detected was a decrease in the height of the susceptibility maximum over time for all MNPs and in both organs, indicating the disappearance of the particles over time. In addition, an increase in the signal corresponding to the presence of ferritin was observed in the spleen samples. As well as the decrease in the maximum height associated with the particles over time, it was interesting to evaluate possible changes in the location and temperature of such maxima. A change in the shape or temperature location of the MNP’s signal in the spleen and liver was observed for all the particles (Additional file 1: Fig. S9 and S10), which was also consistent with the continued degradation of the particles over time. In general, a variation of the temperature corresponding to the maximum of susceptibility was seen with time for the three types of coatings. This variation was greater in the liver with respect to that observed in the spleen, indicating stronger or faster degradation of the MNPs. The data suggested that the liver broke down the particles to a smaller average size over the same time.

Regarding the temperature location of the different susceptibility maxima, the highest temperatures were observed in the spleen for DEX and APS coated MNPs, while for DMSA-MNPs these temperatures were similar in the liver and spleen. Hence, APS and DEX coated MNPs appear to agglomerate more in the spleen [65] than those coated with DMSA. Furthermore, although the amount of DMSA coated MNPs decreased over time in the spleen, the width of the susceptibility signal did not vary as much as with the other particles, suggesting that their particle size distribution was better maintained over time. This could be due to the particles remaining in the spleen being excreted, reducing the number of intact particles in this organ or alternatively, the MNPs were not degraded concomitantly but rather, particle by particle. We consider that their translocation elsewhere from spleen is less probable.

### Evaluation of the MNP concentration over time as an indication of their partial or total degradation in the liver and spleen

To evaluate the degradation of MNPs, the variation in the iron species was quantified from the AC magnetic susceptibility measurements, as described previously [42, 43, 92]. The AC magnetic susceptibility was determined using the sample magnetic moment under an alternating magnetic field at different temperatures, presenting an in-phase or real component (χ') and an out-of-phase or imaginary component (χ"). In a biological sample, all the magnetic species present can contribute to the AC magnetic susceptibility, yet the MNPs are the only species that significantly contribute to χ" [92]. The typical signal of the iron oxide MNPs used for biomedical applications in the out-of-phase susceptibility component has the form of a maximum, and the temperature location of this maximum depends on the size distribution and aggregation of the particles. In addition, the height of this maximum is a surrogate indicator of the number of particles in the sample. This characteristic is very useful to study the presence of NPs in biological samples. Therefore, the amount of iron corresponding to the NPs and to ferritin was quantified from the out-of-phase susceptibility data, while the amount of paramagnetic iron ions in each tissue was quantified from the in-phase susceptibility when this signal was observed [43, 93].

**Fig. 9:**
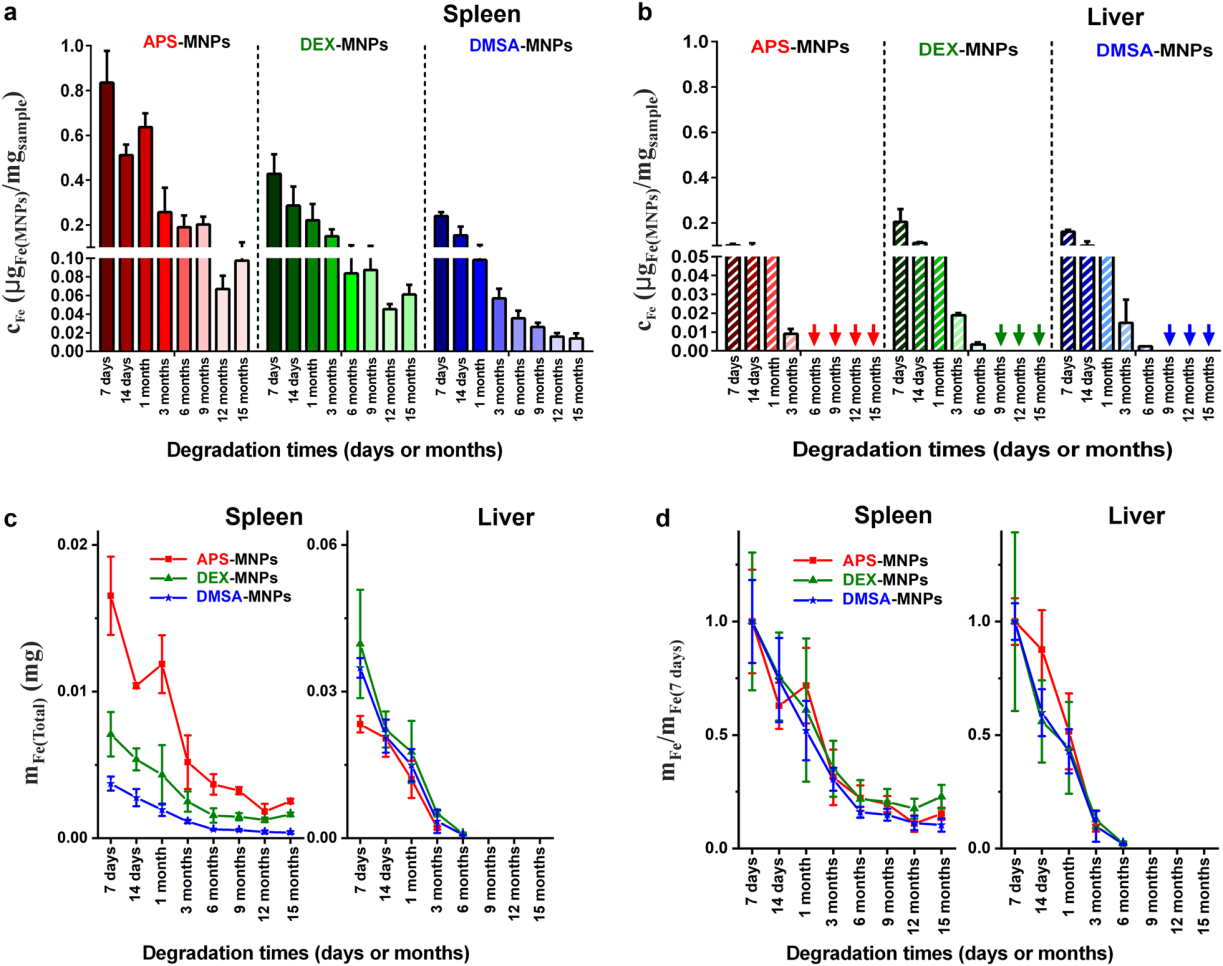
Quantification of the nanoparticles that accumulate in the liver and spleen over time. **a**, **b** After obtaining the magnetic susceptibility values from the spleen and liver, the amounts of the nanoparticles present after their administration was calculated, defining the degradation times of the three types of MNPs. For this, the susceptibility curves of each sample were compared with the known MNP standards, calculating the results per unit mass of tissue. **c** The total iron mass in the spleen and liver was quantified at different degradation times, obtaining the iron mass values from the out-of-phase magnetic susceptibility measurements of the complete spleen and part of the liver. The total iron mass in the liver was estimated from the total mass of this organ. The total iron content in the form of particles was similar in the liver and spleen for the APS-MNPs, whereas for the DEX- and DMSA-MNPs the total iron mass was larger in the liver than the spleen. **d** The total iron mass determined at different times (m_Fe_) and normalized to the estimated mass at the first time point (${\mathrm{m}}_{{\mathrm{Max}}_{7\mathrm{days}}}$) for the liver and spleen. This relationship allowed the degradation speed of the particles in the different organs to be better visualized since the effect of the initial accumulation of particles in the tissues was normalized. The data are shown as the means ± SD (n = 3)

The number of particles was estimated in the spleen and liver for up to 15 months and in general, the iron concentration in the form of particles with the same coating was always higher in the spleen than in the liver at the same time point (see Fig. 9a, b). Nevertheless, as the liver is a much larger organ than the spleen, the total iron content in the form of particles was distinct in the liver and spleen (Fig. 9c). In terms of MNPs coated with APS, the total iron mass corresponding to MNPs was similar in the spleen and liver, while for MNPs coated with DEX or DMSA the MNP iron mass in the liver was 5 or 9 times greater than in the spleen for approximately 7–14 days. The concentration of particles in both tissues decreased systematically over time, suggesting that the MNPs accumulated in these organs were degraded. In the long term, it was interesting that 9 months after their administration none of the three types of MNPs were detected in liver tissues.

To compare the rate of degradation between these organs without taking into account the effect of the initial accumulation of particles, the total iron mass was determined at different times and normalized to the iron mass quantified at 7 days for each organ. In general, degradation in the liver was faster than in the spleen and similar for the three types of MNPs in this tissue (Fig. 9d). There was no evidence that the speed of MNP degradation in the liver was affected by the type of coating. However, there were small differences in the time when MNPs were no longer detected, although this was probably due to the fact that the MNPs coated with DEX and DMSA initially accumulated more intensely than the APS-MNPs, requiring slightly longer for their complete degradation. Moreover, the differences in the initial accumulation may be related to the different MNP coatings. The time for complete clearance of the MNPs in the liver was consistent with the immunohistochemical analysis of these tissues, which suggested the faster clearance of DMSA coated MNPs in the liver than those coated with APS or DEX. Previous studies into the long-term in vivo fate of gold/iron oxide heterostructures (NHs) showed that their accumulation in the liver was greater in NHs coated with an amphiphilic polymer (PC-NHs) than those with poly(ethylene glycol), PEG-NHs [36]. This is due to the effect of some coatings like PEG in reducing opsonization by macrophages, which enhances the circulation time of the NPs [94, 95]. By contrast, the negatively charged amphiphilic polymer is not as effective in preventing macrophage uptake in the liver and spleen [27, 36, 59].

Other studies that monitored the bioassimilation of empty copper sulfide NPs (CuS-NPs) or NPs with a flower-like core of iron oxide (iron oxide@CuS-NPs) finally coated with PEG, showed their accumulation mainly in the liver and spleen following i.v. administration to 6-week-old Balb/C mice [96]. Regarding the degradation of these particles up to 6 months post-administration, they appeared to remain intact within the liver and spleen in TEM images for up to 7 days post-administration. However, 3 months after administration no intact hybrid particles were detected, although structures similar to ferritin were detected that were indicative of their degradation [96]. Ferritin also appeared here, probably reflecting the degradation of the MNPs although at times that differed from those indicated previously, which might vary for different reasons including the particle coating.

By contrast, slower degradation was seen in the spleen than in the liver and even 15 months after their administration, the three types of MNPs could be detected there. The degradation speed was similar for the three coatings in the spleen (Fig. 9d). Recent studies carried out on 17 commercial particles with different physicochemical properties (size, coating and surface charge) showed faster degradation of smaller particles and for those with a negative Z-potential, indicating that the type and structure of the coating strongly influences MNP degradation [27]. Here, we included an additional parameter that should be taken into account in future degradation studies, the amount of particles that initially accumulate within an organ, as this may be a key parameter to determine the time for complete particle clearance in an organ. The differences in the rate of degradation between APS and DMSA coated MNPs could be explained primarily by their surface charge, which dictates how quickly MNPs are trapped by macrophages. Our results suggest new directions to control NP degradation by indicating that the composition of the synthetic surface affects the residence time of NPs in the body.

### Analysis of other iron-containing species in the liver and spleen

**Fig. 10:**
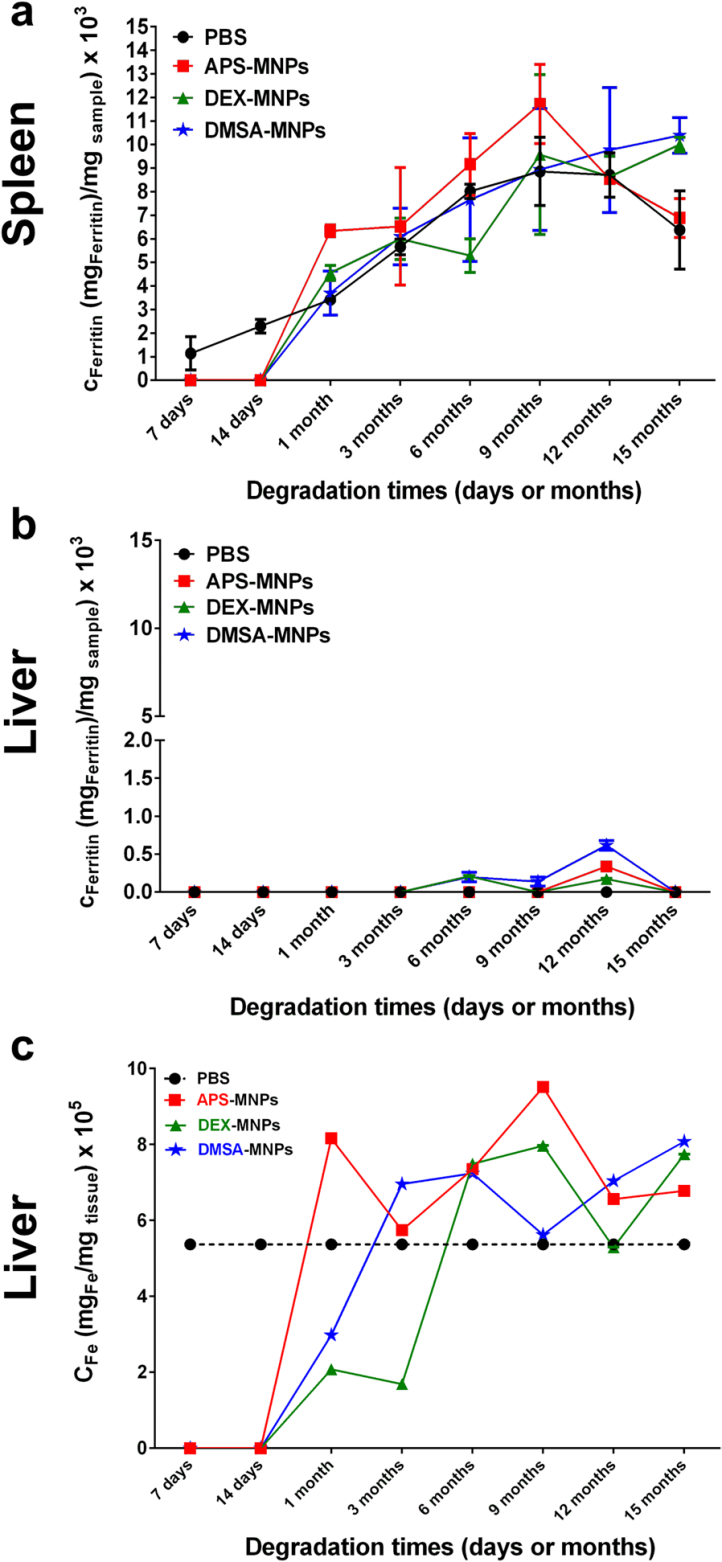
Ferritin and paramagnetic iron quantification over time as an indicator of MNP degradation. **a** Spleen and **b** liver ferritin concentration from 7 days after the last administration to 15 months. **c** Liver paramagnetic iron concentrations over time quantified from the in-phase susceptibility data. The data are shown as the means ± SD (n = 2)

The kinetics of ferritin accumulation were followed through a quantitative analysis of the iron concentration stored in the form of this protein (see Fig. 10). Ferritin presents a superparamagnetic behavior at room temperature and a characteristic out-of-phase magnetic susceptibility maxima at around 8–10 K, in the same AC field conditions as those used here [45]. A reference ferritin from mouse liver was used for the quantitative analysis and in general, there was a trend towards higher concentrations of iron stored in the form of ferritin in all the spleens studied over time, including the controls. This may be explained by the natural iron accumulation in the spleen over the life time of the animals [55, 97].

At the shortest time points studied, 7 and 14 days after administration, no ferritin was detected in the spleens of any of the treated groups, whereas a small amount was detected in the controls (Additional file 1: Fig. S11). This may be due to the strong signal from the particles masking the smaller ferritin contribution (Fig. 8). Between 1 and 9 months, the signal corresponding to ferritin for the DMSA- and DEX-MNPs was very similar to the controls, whereas the APS-MNPs produced slightly more ferritin than the controls in that period of time. The difficulty in assessing iron accumulation in the form of ferritin is a consequence of the small amount of iron in the form of particles that accumulate in the organs (0.3–0.8 μg_Fe-MNPs_/mg_tissue_) and that is potentially degraded into ferritin relative to the amount of endogenous ferritin (9 μg_Fe-ferritin_/mg_tissue_). This difference (> tenfold) hinders accurate comparisons given the variability in the animals. At the last time point, 15 months, the amount of ferritin in the mice administered DMSA- and DEX-MNPs was higher than in the controls and in those administered APS-MNPs (Fig. 10a).

A completely different behavior was observed in the liver and no ferritin signal was detected in control organs at any point in the experiment. In the treated mice, it was possible to detect a small signal corresponding to ferritin in some of the liver samples long after MNP administration (6 months in the mice administered DMSA and DEX coated MNPs, and at 12 months in the APS-MNP mice). This behavior was consistent with the degradation of MNPs and the partial accumulation of ferritin. At the last time point measured, 15 months after MNP administration, no ferritin signal was detected, which probably reflects the complete clearance of MNPs as no sign of the MNPs was detected 9 months after their administration (Fig. 10b).

After quantifying the MNPs accumulated in the liver from the out-of-phase susceptibility, the paramagnetic iron that corresponds to iron ions not forming part of any mineral species was quantified from the in-phase susceptibility. In general, a similar amount of paramagnetic iron was observed (Fig. 10c) independent of the time after administration or the particle coating (Additional file 1: Fig. S11), indicating that such paramagnetic species were probably part of the basal iron species and that they were not formed as a result of MNP degradation. In the case of iron oxides, dissolution and recrystallization to form other magnetic nanoparticles has only been proved for the very specific case of stem cells [98]. In general, given the iron metabolism pathways present in the organism, the iron atoms released from the particles become part of the body iron, mineralizing in the form of ferritin, as observed here or being uptaken by other proteins related to the iron storage and transport [75].

In summary, after a period in the blood, MNPs injected intravenously accumulate in the spleen and liver, being taken up by macrophages in these organs. The amount of particles that accumulated in the different tissues is affected by their coating, a key parameter to understand the time needed for the complete clearance of the particles from the different organs. MNPs were degraded at different rates depending on the organ, degrading faster in the liver than in the spleen. In particular, the MNPs had been completely degraded in the liver after 15 months while some particles still remained in the spleen. Depending on the residence time in the organism required, a tailored coating could be designed to fulfil the needs of any future application.

## Conclusions

Studying the biotransformation of MNPs after their internalization by macrophages in the organs where they accumulate and how their degradation occurs is crucial to improve their performance in biomedical applications. Here we show that three MNPs with the same iron oxide core but with different coatings have different degradation rates in vitro and in vivo. The biodistribution studies in vivo show that APS, DEX and DMSA coated MNPs accumulate in the liver and spleen, regardless of their coating, although the proportions that accumulate in each organ differ depending on the coating: APS-MNPs accumulate more in the spleen while DMSA-MNPs accumulate more in the liver. In vitro degradation studies show that the APS and DMSA coated MNPs degraded at a similar rate in RAW 264.7 cells (a circulating macrophage-like cell line), in which the DEX-MNPs degraded more slowly. By contrast, DMSA-MNPs were degraded more rapidly in the NCTC1469 cell line (a mouse liver-derived macrophage-like cell line), followed by APS-MNPs and finally DEX-MNPs. These results suggest that the endolysosomal degradation rate of MNPs varies depends on the type of MNP coating and the macrophages or organ in which they accumulate. There were no signs of long-term liver toxicity and the circulation time in the blood of each type of MNPs was less than 7 days. In the liver, the speed of degradation was similar for the three different coatings, although the differences in their accumulation determined the time needed for their complete clearance. Finally, i*n vivo* degradation studies showed that the degradation speed of MNPs differed in the organ in which they were located, and it was faster in the liver than the spleen. This study allows us to predict the biodistribution and total degradation of the particles based on their physicochemical properties, mainly their coating and surface charge, for their future biomedical application in antitumor treatments.

### Supplementary Information

**Additional file 1**: **Fig. S1**. Physicochemical characterization of MNPs. (a) TEM images of iron oxide coated APS-, DEX-and DMSA-MNPs. (b) Nanoparticle size distributions after coating with APS, DEX and DMSA, as determined by TEM. (c, d) The hydrodynamic size and Z potential of the APS, DEX and DMSA coated MNPs at 24 h as determined by DLS. The data shown for means ± SD corresponding to the hydrodynamic size of the MNPs were obtained by calculating the mean value of hydrodynamic size measured in intensity distribution for three measurements made with this batch of MNPs. (e) Magnetization curve at RT for the MNPs showing their superparamagnetic behavior. (f) Magnetic susceptibility of APS-, DEX- and DMSA-MNPs diluted in agar. Scale bars 50 nm and 20 nm. **Fig. S2**. Evaluation of the blood circulation time of MNPs. (a) ICP-OES analysis of iron content in blood samples from PBS (Control), APS-, DEX- or DMSA-MNP-treated mice 7 days post-administration. The data are shown as the mean ± SD (n = 3). (b, c) Analysis of the presence of APS-MNPs in blood determined by AC magnetic susceptibility 7 (b) and 14 (c) days after the last administration of the APS-MNPs. The in-phase (real, χ′) and out-of-phase (imaginary, χ′′- inset inside of the χ′ profile) components of the AC magnetic susceptibility measurements are shown. **Fig. S3**. AC magnetic susceptibility of the organs of PBS-treated mice 7 days after they received the last dose of PBS. Temperature dependence of the AC magnetic susceptibility: a) in-phase and b) out-of-phase components of tissues from mice treated with PBS at 7 days post-administration. **Fig. S4**. Analysis of protein corona formation dynamics on APS-, DEX- and DMSA-MNPs in DMEM with 10% MS. Hydrodynamic size and Z-Potential over time as determined by DLS: (a) APS-MNPs, (b) DEX-MNPs and (c) DMSA-MNPs. Control sample, MNPs incubated in DMEM without MS; Corona sample, MNPs incubated in DMEM with 10% MS to allow PC formation. In both cases, the DLS measurements were performed in triplicate. **Fig. S5**. Magnetically isolated endolysosomes containing MNPs. (a) Endolysosomal Lamp 1 marker found in endolysosomes containing MNPs assessed in Western blots. (b) Quantification of Lamp1 levels by densitometry of the bands obtained in Western blot using the Image J software. **Fig. S6**. Viability of macrophage cells treated with different concentrations of APS-, DEX- and DMSA-MNPs. Cell viability of RAW264.7 (a) and NCTC1469 cells (b). Concentration dependent cytotoxic effects of MNPs evaluated with the PrestoBlue assay after a 24 h incubation. The data are the mean ± SD of three independent experiments in both analyses. **Fig. S7**. TEM images of MNPs before and after their internalization in endolysosomes in RAW 264.7 and NCTC1469 macrophagic cells. These images were analyzed to determine the decrease in the size of the iron oxide core as indicative of intracellular degradation. Scale bar 20 nm. **Fig. S8**. Cellular iron concentrations in macrophage cells after APS-, DEX- and DMSA-MNP uptake. (a) Quantification of the iron concentration in RAW 264.7 cells by ICP-OES. (b) (a) Quantification of the iron concentration in NCTC1469 cells by ICP-OES. The data are shown as the mean ± SD (n = 3) and the iron concentration in both cell types was compared by a one-way analysis of variance (ANOVA) and Tukey’s multiple tests. The asterisks indicate statistically significant differences in iron concentration: ns – no significant differences, *p <0.05, **p <0.01, ***p <0.001, and **** p <0.0001. **Fig. S9**. Temperature dependence of the in-phase and out-of-phase susceptibility scaled to the maximum associated with the presence of particles in the spleen. These plots help visualize the change in position of the AC magnetic susceptibility maximum over time, indicating particle transformation. (a) APS-MNPs, (b) DEX-MNPs and (c) DMSA-MNPs. The in-phase (real, χ′ - Top) and out-of-phase (imaginary, χ′′ - Bottom) component of the AC magnetic susceptibility measurements are shown. **Fig. S10**. Temperature dependence of the in-phase and out-of-phase susceptibility scaled to the maximum associated with the presence of particles in the liver. These plots help visualize the change in position of the AC magnetic susceptibility maximum over time indicating particle transformation. (a) APS-MNPs, (b) DEX-MNPs and (c) DMSA-MNPs. The in-phase (real, χ′ - Top) and out-of-phase (imaginary, χ′′ - Bottom) component of the AC magnetic susceptibility measurements are shown. **Fig. S11**. Evolution of the ferritin and paramagnetic ion signals in PBS-treated mice measured by AC magnetic susceptibility at different times. The magnetic susceptibility showed a paramagnetic signal in the in-phase magnetic susceptibility component in the liver, although no paramagnetic contribution was observed in the spleen. In addition, a ferritin signal in the out-of-phase magnetic susceptibility component that increased over time was observed in the spleen but not in the liver. The in-phase (real, χ′ - Top) and out-of-phase (imaginary, χ′′ - Bottom) component of the AC magnetic susceptibility measurements of the liver (a) and spleen (b) are shown.

## Data Availability

All data generated or analyzed during this study are included in this published article and its Additional files.
