# Supplementary material for: Different coatings on magnetic nanoparticles dictate their degradation kinetics in vivo for 15 months after intravenous administration in mice
Source: J Nanobiotechnology. 2022 Dec 28;20:543. doi: 10.1186/s12951-022-01747-5 (PMC9795732; doi:10.1186/s12951-022-01747-5)
Supplement: Supplementary file 1 — Additional file 1: Fig. S1. Physicochemical characterization of MNPs. (a) TEM images of iron oxide coated APS-, DEX-and DMSA-MNPs. (b) Nanoparticle size distributions after coating with APS, DEX and DMSA, as determined by TEM. (c, d) The hydrodynamic size and Z potential of the APS, DEX and DMSA coated MNPs at 24 h as determined by DLS. The data shown for means ± SD corresponding to the hydrodynamic size of the MNPs were obtained by calculating the mean value of hydrodynamic size measured in intensity distribution for three measurements made with this batch of MNPs. (e) Magnetization curve at RT for the MNPs showing their superparamagnetic behavior. (f) Magnetic susceptibility of APS-, DEX- and DMSA-MNPs diluted in agar. Scale bars 50 nm and 20 nm. Fig. S2. Evaluation of the blood circulation time of MNPs. (a) ICP-OES analysis of iron content in blood samples from PBS (Control), APS-, DEX- or DMSA-MNP-treated mice 7 days post-administration. The data are shown as the mean ± SD (n = 3). (b, c) Analysis of the presence of APS-MNPs in blood determined by AC magnetic susceptibility 7 (b) and 14 (c) days after the last administration of the APS-MNPs. The in-phase (real, χ′) and out-of-phase (imaginary, χ′′- inset inside of the χ′ profile) components of the AC magnetic susceptibility measurements are shown. Fig. S3. AC magnetic susceptibility of the organs of PBS-treated mice 7 days after they received the last dose of PBS. Temperature dependence of the AC magnetic susceptibility: a) in-phase and b) out-of-phase components of tissues from mice treated with PBS at 7 days post-administration. Fig. S4. Analysis of protein corona formation dynamics on APS-, DEX- and DMSA-MNPs in DMEM with 10% MS. Hydrodynamic size and Z-Potential over time as determined by DLS: (a) APS-MNPs, (b) DEX-MNPs and (c) DMSA-MNPs. Control sample, MNPs incubated in DMEM without MS; Corona sample, MNPs incubated in DMEM with 10% MS to allow PC formation. In both cases, the DLS measurements were perform [file 12951_2022_1747_MOESM1_ESM.pdf]

Different coatings on magnetic nanoparticles dictate their degradation kinetics *in vivo* for 15 months after intravenous administration in mice

Yadileiny Portilla[1#], Yilian Fernández-Afonso[2#], Sonia Pérez-Yagüe[1], Vladimir Mulens-Arias[1$], María del Puerto Morales[3], Lucía Gutiérrez[2*] and Domingo F. Barber[1*]

[1] Department of Immunology and Oncology and the NanoBiomedicine Initiative, Centro Nacional de Biotecnología (CNB)/CSIC, Darwin 3, Cantoblanco, 28049 Madrid, Spain.

[2] Departamento de Química Analítica, Instituto de Nanociencia y Materiales de Aragón (INMA), Universidad de Zaragoza, CSIC and CIBER-BBN, 50018 Zaragoza, Spain.

[3] Department of Energy, Environment and Health, Instituto de Ciencia de Materiales de Madrid (ICMM-CSIC), Sor Juana Inés de la Cruz 3, 28049 Madrid, Spain.

[$]Current address: Integrative Biomedical Materials and Nanomedicine Laboratory, Department of Medicine and Life Sciences (MELIS), Pompeu Fabra University, Carrer Doctor Aiguader 88, 08003 Barcelona, Spain.

Author contributions:

[#] Y.P. and Y.F.A. contributed equally to this work.

Corresponding authors:

[*]E-mail: dfbarber@cnb.csic.es (D.F.B).

[*]E-mail: lu@unizar.es (L.G)

Additional file 1. Materials and Methods

Western blots to verify endolysosome isolation

After the fractions enriched in endolysosomes were obtained they were lysed at 4 °C for 45 min in lysis buffer (1% Triton-X 100, 50 mM Tris, 150 mM NaCl, 5 mM NaF, 1 mM sodium orthovanadate, 1 mM PMSF, 1 mM EDTA, 1 μg/ml aprotinin, 1 μg/ml leupeptin and 2 nM okadaic acid). The lysate was clarified by centrifugation and an aliquot of total protein from each sample (40 μg, as measured with the Micro BCA protein assay kit: Pierce) was loaded onto polyacrylamide gels and resolved by SDS-PAGE (10%). The proteins were then transferred to a 0.2 μm pore PVDF nitrocellulose membrane (BioRad), which was blocked with 10% w/v non-fat milk in Tris buffered saline (TBS) and then probed with specific primary antibodies against Lamp1 (1D4B, DSHB). The membranes were then washed and incubated for 1 h at RT with an HRP-conjugated anti-rat immunoglobulin secondary antibody diluted 1:1000 in TBS/0.05% Tween 20 (TBS-T), and then washed extensively TBS-T. Antibody binding was visualized with ECL Western Blotting Detection Reagent (RPN2106; Amersham) for up to 5 min, then quantified and analyzed with ImageJ software (US National Institutes of Health).

PrestoBlue cytotoxicity assay

Cell viability was analyzed with the PrestoBlue assay (Invitrogen, Thermofisher Scientific, Madrid, Spain). The cells were cultured for 24 h in 96-well plates (2×10^4 cells/well RAW 264.7 cells and 5×10^4 NCTC1469 cells) and then, exposed for 24 h to APS, DEX or DMSA coated MNPs at different concentrations (from 0 to 500 μg Fe/ml). PrestoBlue (1:10) was added to each well for 1 h and the fluorescence was measured (530 nm excitation, 590 nm emission), expressing cell viability as the fluorescence of the treated cells relative to the untreated cells and presenting the results as the mean values from three experiments.

Iron quantification in RAW 264.7 and NCTC1469 cells by ICP-OES

RAW 264.7 and NCTC1469 cells were seeded in a 6-well plate at a density of 2×10^5 cells per well, and incubated for 24 h at 37 °C. APS, DEX or DMSA coated MNPs were then added to the cells at different concentrations (50, 125 and 250 μg/ml) and incubated in identical conditions for 24 h. After incubation, the non-internalized particles were removed by washing the cells three times with PBS, and the cells were then harvested

and counted in a Neubauer chamber. The samples were digested in 1 ml of 37% HCl (Merck) for 1 h at 90 °C and then made up to a final volume of 10 ml with distilled water. The amount of iron relative to the number of cells in each sample was measured using ICP-OES (Perkin Elmer-2400) at the Chemical Analysis Service of the ICMM-CSIC.

Iron quantification in blood by ICP-OES

The iron was quantified in the blood at different times after MNP administration by ICP-OES analysis, as indicated for the cell lysates (see above). The mice administered PBS alone were used as controls to determine the basal iron concentration in each sample. The amount of iron was expressed relative to the weight of the sample determined previously.

Corona formation in mouse serum

Protein corona (PC) formation was studied by incubating MNPs (APS, DEX or DMSA coated MNPs) at a concentration of 125 μg Fe/ml for different times (0, 1, 3, 5, 10, 24, 48, 72 h) in DMEM medium supplemented with 10% mouse serum (MS). As a negative control the same experimental conditions were used without MS. The hydrodynamic size was measured following the protocol described for MNP physicochemical characterization. To analyze the surface charge, the Z potential was measured at pH 7 using the NanoSizer ZS (Malvern).

Additional file 1. Results

Physicochemical characterization of MNPs

Figure S1 summarizes the main physicochemical characteristics of the MNPs used during this study and characterized previously(1, 2).

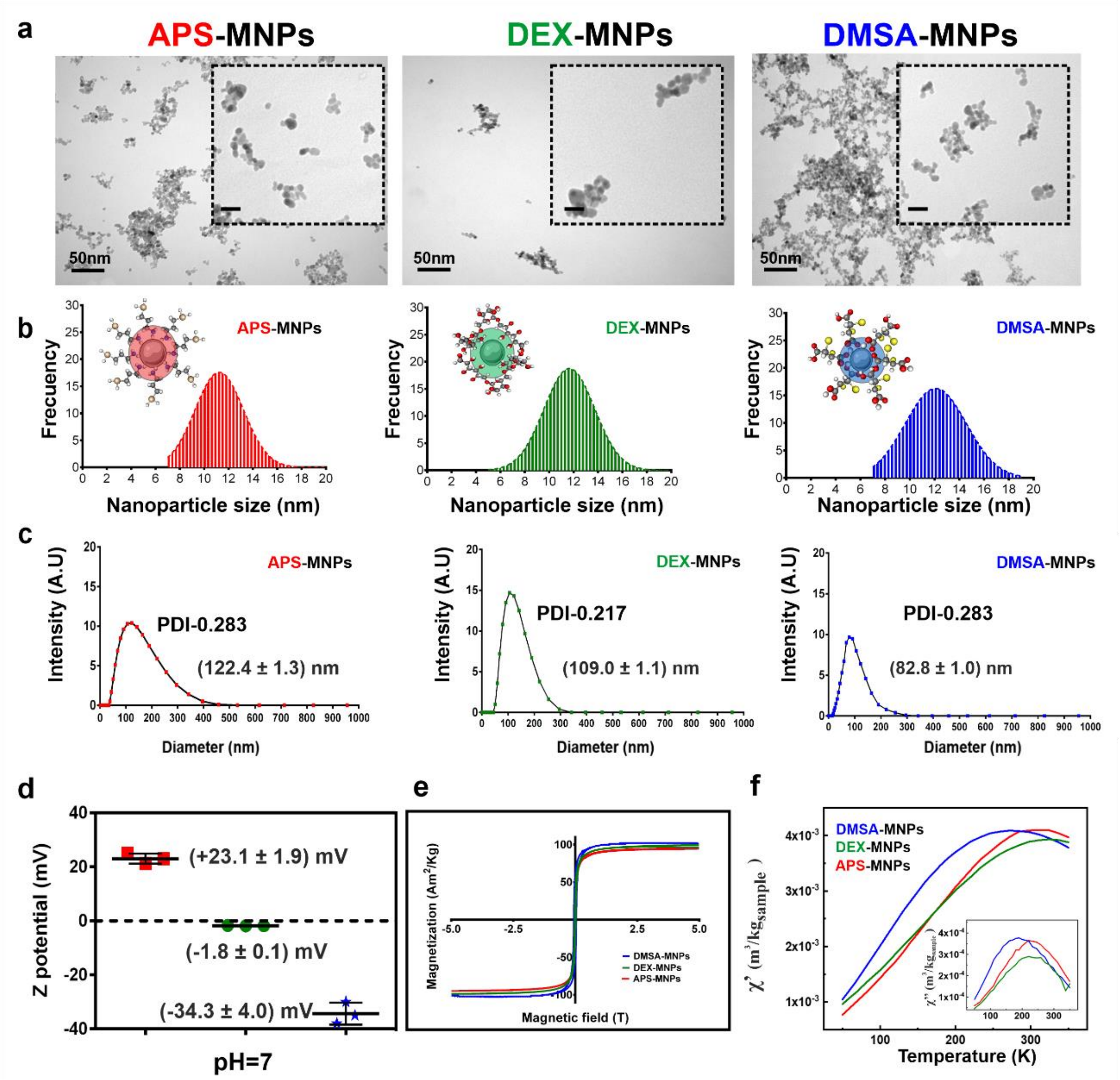

Fig. S1. Physicochemical characterization of MNPs. (a) TEM images of iron oxide coated APS-, DEX- and DMSA-MNPs. (b) Nanoparticle size distributions after coating with APS, DEX and DMSA, as determined by TEM. (c, d) The hydrodynamic size and Z potential of the APS, DEX and DMSA coated MNPs at 24 h as determined by DLS. The data shown for means ± SD corresponding to the hydrodynamic size of the MNPs were obtained by calculating the mean value of hydrodynamic size measured in intensity distribution for three measurements made with this batch of MNPs. (e) Magnetization curve at RT for the MNPs showing their superparamagnetic behavior. (f) Magnetic susceptibility of APS-, DEX- and DMSA-MNPs diluted in agar. Scale bars 50 nm and 20 nm.

Evaluation of the blood circulation time of MNPs

To assess whether the particles were still circulating in the bloodstream 7 days after the last dose of MNPs was administered, two experimental approaches were followed: ICP-OES analysis, which allowed us to quantify the total amount of iron in the blood; and AC

magnetic susceptibility that determined the presence of MNPs in the blood. The levels of iron detected by ICP-OES in the blood of mice treated with MNPs were lower than those of the control group not exposed to MNPs (10.57 ± 1.24 µg/mg of sample). In mice administered APS coated MNPs, the iron content detected was 7.55 (± 3.23) µg/mg of sample, while in those treated with DEX- and DMSA-MNPs the blood iron concentrations were 4.55 (± 0.41) µg/mg of sample and 3.90 (± 0.49) µg/mg of sample, respectively (**Fig. S2a**). Finally, a blood sample was analyzed for AC magnetic susceptibility in mice treated with APS-MNPs at 7 and 14 days, which was the type of coating where the greatest amount of iron was observed in the blood by ICP-OES analysis, and the results confirmed that no particles were found in the bloodstream after 7 days (**Fig. S2b and S2c**).

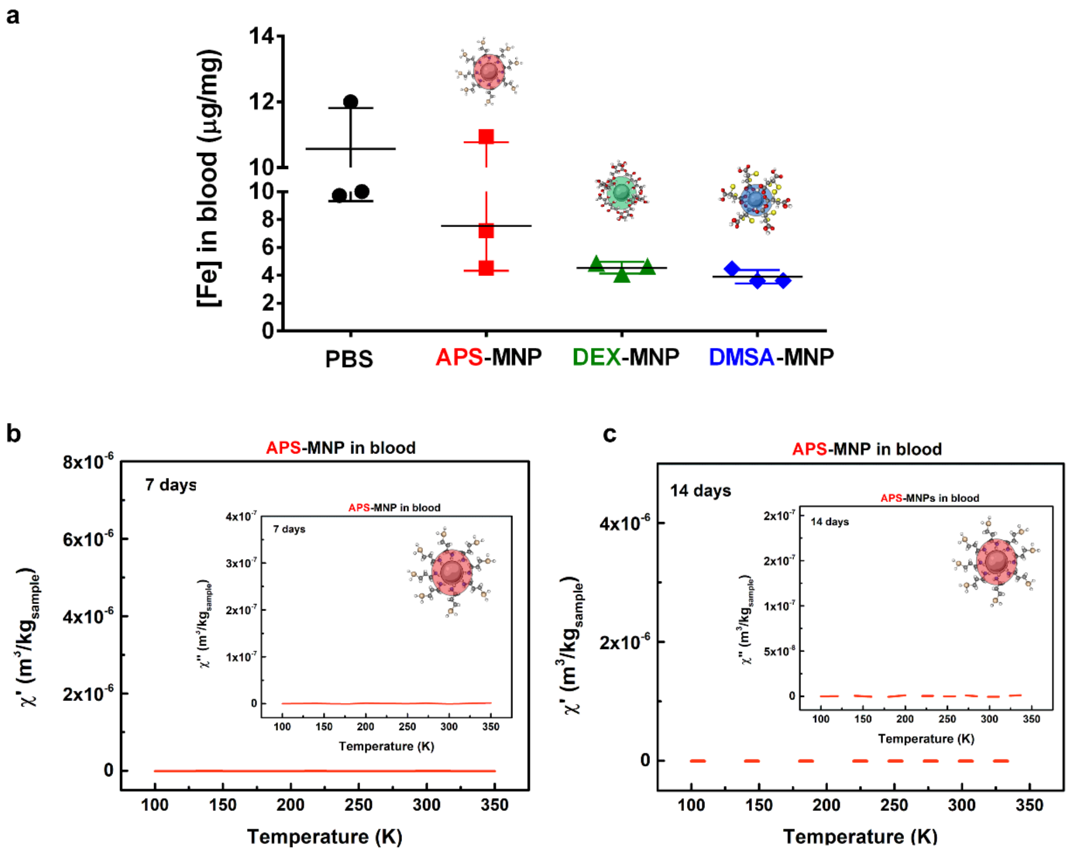

Fig. S2. Evaluation of the blood circulation time of MNPs. (a) ICP-OES analysis of iron content in blood samples from PBS (Control), APS-, DEX- or DMSA-MNP-treated mice 7 days post-administration. The data are shown as the mean ± SD (n = 3). (b, c) Analysis of the presence of APS-MNPs in blood determined by AC magnetic susceptibility 7 (b) and 14 (c) days after the last administration of the APS-MNPs. The in-phase (real, χ') and out-of-phase (imaginary, χ''- inset inside of the χ' profile) components of the AC magnetic susceptibility measurements are shown.

AC magnetic susceptibility measurement of the PBS-treated mouse organs

AC magnetic susceptibility was measured 7 days after the last PBS dose was administered to control mice to detect any magnetic signal in these mice in the temperature range studied. These measurements were carried out under the same conditions as those used for the treated mice.

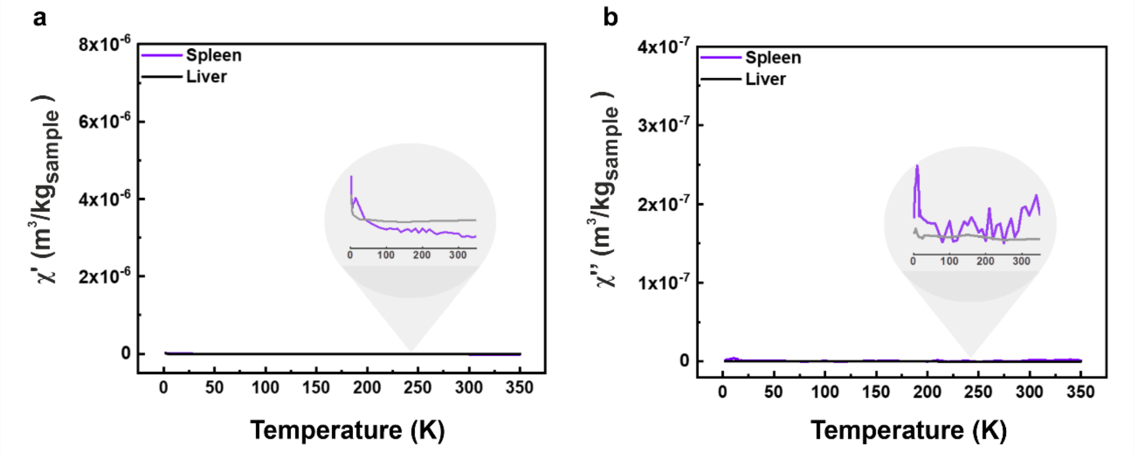

Fig. S3. AC magnetic susceptibility of the organs of PBS-treated mice 7 days after they received the last dose of PBS. Temperature dependence of the AC magnetic susceptibility: a) in-phase and b) out-of-phase components of tissues from mice treated with PBS at 7 days post-administration.

As expected, no signal was detected in the temperature range where the AC magnetic susceptibility maximum for the particles appears. A small maximum in the out-of-phase magnetic susceptibility was evident around 8 K in the spleens corresponding to the ferritin signal. Ferritin in spleens was expected to occur due to the natural process of iron accumulation in this organ over the animal's life time.

Evaluation of corona formation in mouse serum

In order to evaluate the effects of different coatings on PC formation, APS-, DEX- or DMSA-MNPs were incubated in DMEM medium supplemented with 10% MS for different periods of time (0, 1, 3, 5, 10, 24, 48 and 72 h). As a control, APS-, DEX- or DMSA-MNPs were incubated in DMEM without MS for the same periods of time. PC formation was then characterized at each time point by measuring the changes in hydrodynamic size and variation in the Z-potentials of the different coated MNPs. The hydrodynamic size and Z-potential values were measured by dynamic light scattering (DLS: **Fig. S4**).

The PC formation dynamics graphics showed that coated MNPs reached their maximum hydrodynamic size at 10 h of incubation, after which their hydrodynamic size decreased until it stabilized at approximately 24 h. The maximum hydrodynamic size observed in

each case was 2630 nm for APS-MNPs, 683 nm for DEX-MNPs and 178 nm for DMSA-MNPs. This maximum peak in the hydrodynamic size of coated MNPs could be explained by the presence of a soft unstable corona over the more stable inner corona. The stabilization in size observed at 24 h would be the consequence of the dynamic exchange of proteins between the medium and the corona at the surface of the coated MNPs reaching an equilibrium, which would give rise to the formation of a stable corona.

The Z-potentials were always negative when the three types of coated MNPs were incubated in medium with 10% MS. These differences in Z-potentials were due to the formation of a PC in all conditions where FBS was present in the incubation medium. In the case of APS-MNPs, we observed positive Z-potentials in conditions without MS, while in the presence of FBS the median Z-potentials shifted to negative values. For DEX-MNPs, the Z-potentials in the samples without MS were close to zero, however in the presence of FBS negative Z-potentials were observed. These variations in the Z-potentials could be caused by the proteins in FBS that contributed to the PC adding negative charges, increasing their negative Z-potentials. In the case of DMSA-MNPs, in Z-potentials were negative in both the presence and absence of MS, however they were less negative in the presence of MS, which might be explained by DMSA-MNPs which have a negative Z-potential tending to bind proteins with hydrophobic residues.

The differences in the increase in hydrodynamic size between MNPs with different coatings after incubation in medium with 10% MS could reflect the different surface charges provided by each coating. Comparing the hydrodynamic sizes of the MNPs with different coatings, the largest increase after 24 h was observed in APS-MNPs (from 211 to 1554 nm), an ~7-fold change, while DEX-MNPs increased ~4-fold (from 148 to 586 nm) and in DMSA-MNPs~2-fold (from 83 to 165 nm).

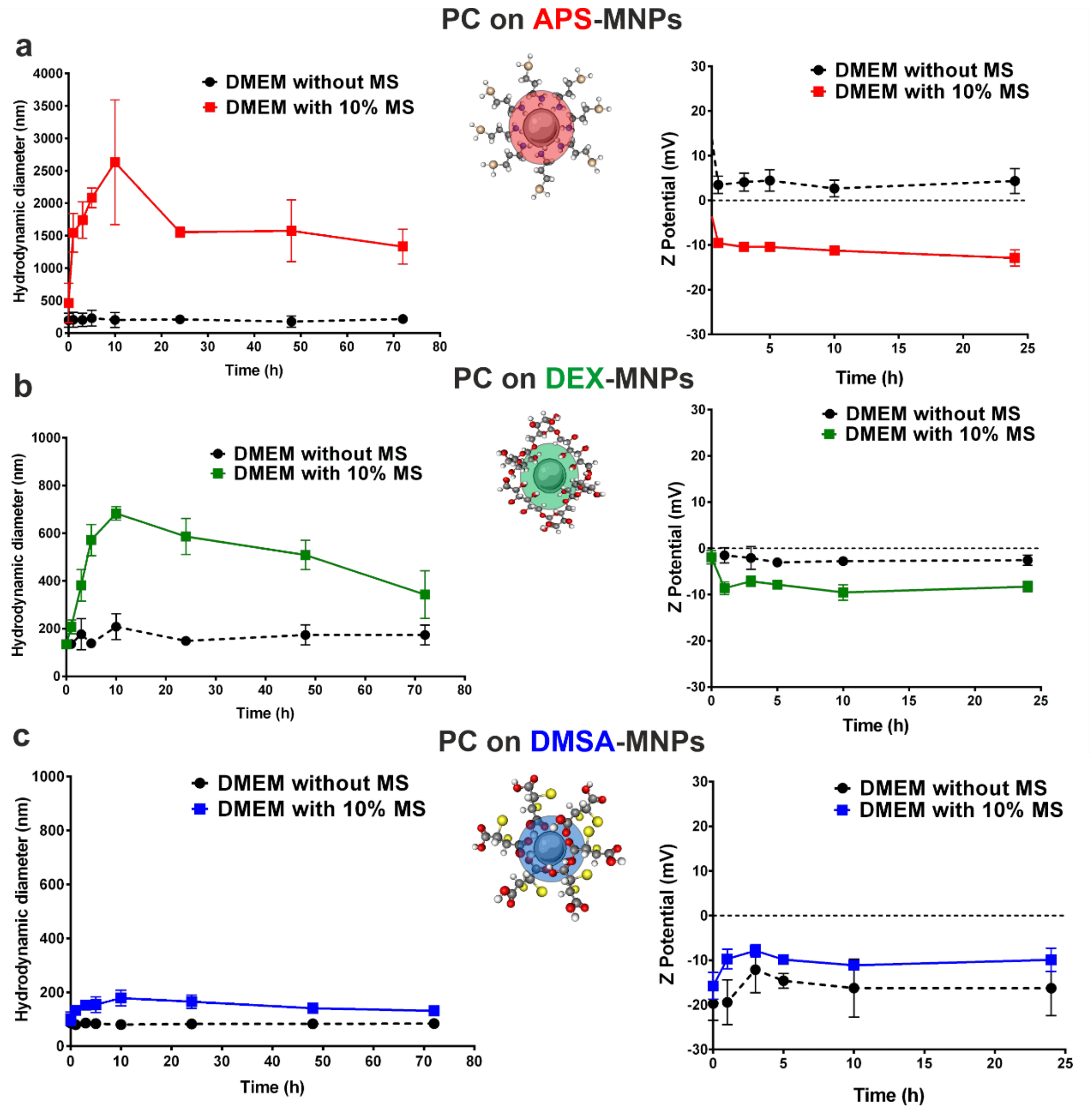

Fig. S4. Analysis of protein corona formation dynamics on APS-, DEX- and DMSA-MNPs in DMEM with 10% MS. Hydrodynamic size and Z-Potential over time as determined by DLS: (a) APS-MNPs, (b) DEX-MNPs and (c) DMSA-MNPs. Control sample, MNPs incubated in DMEM without MS; Corona sample, MNPs incubated in DMEM with 10% MS to allow PC formation. In both cases, the DLS measurements were performed in triplicate.

Western blots to assess MNP-loaded endolysosome enrichment

The vesicles loaded with each of the MNPs offered a profile of the final endolysosomal compartment and only endolysosomes containing MNPs appeared to be isolated, as witnessed by the enrichment of the lysosomal marker Lamp1 (**Fig. S5a and S5b**).

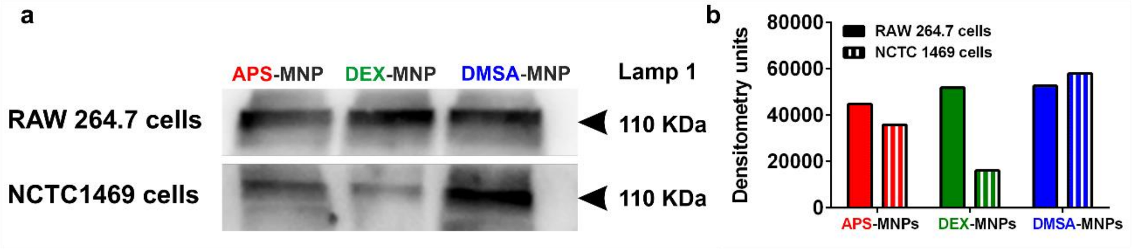

Fig. S5. Magnetically isolated endolysosomes containing MNPs. (a) Endolysosomal Lamp 1 marker found in endolysosomes containing MNPs assessed in Western blots. (b) Quantification of Lamp1 levels by densitometry of the bands obtained in Western blot using the Image J software.

Cell viability studies to determine the optimal concentration of MNPs to isolate MNP-loaded endolysosomes

MNP treatment did not affect RAW 264.7 and NCTC1469 cell viability in the PrestoBlue assays (**Fig. S6**). The fluorescence readings from the PrestoBlue assays of the MNP-treated RAW 264.7 cells indicated a slight increase in the mitochondrial metabolism of these cells. The optimal MNP concentration selected was 125 μg/ml for both cell types.

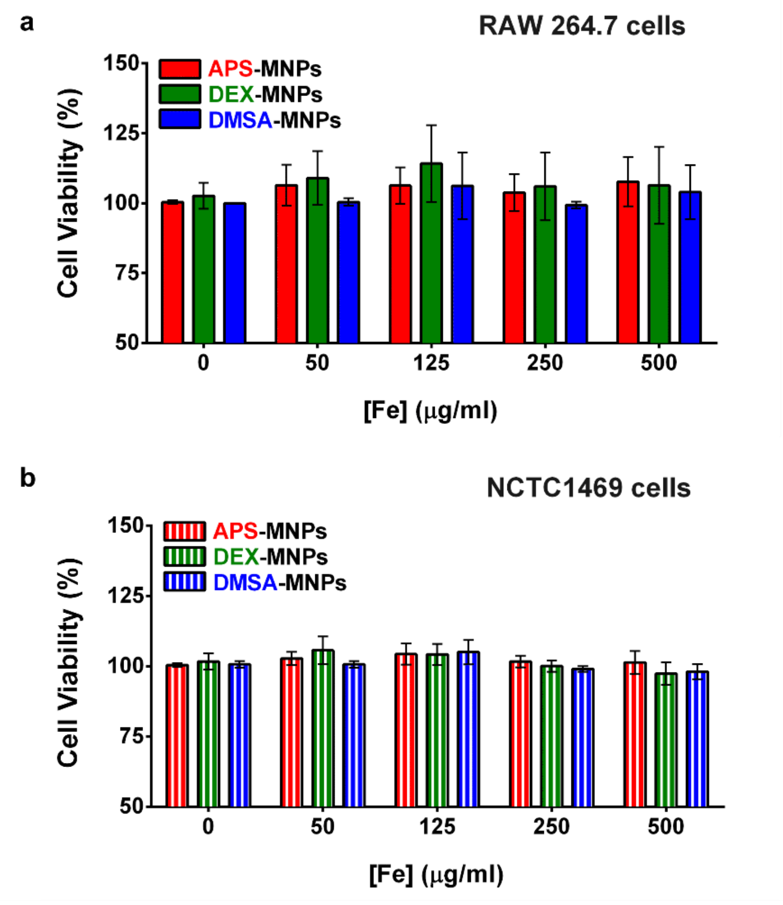

Fig. S6. Viability of macrophage cells treated with different concentrations of APS-, DEX- and DMSA-MNPs. Cell viability of RAW264.7 (a) and NCTC1469 cells (b). Concentration dependent cytotoxic effects of MNPs evaluated with the PrestoBlue assay after a 24 h incubation. The data are the mean ± SD of three independent experiments in both analyses.

TEM images of MNPs isolated from endolysosomes

In the Figure S7 we observed the TEM images of MNPs (APS-, DEX- or DMSA-MNPs) after being incubated for 24 h with RAW 264.7 or NCTC1469 macrophagic cells and

then subcellularly located in endolysosomes. As a control of the size of the iron oxide core, the MNPs resuspended in water are observed.

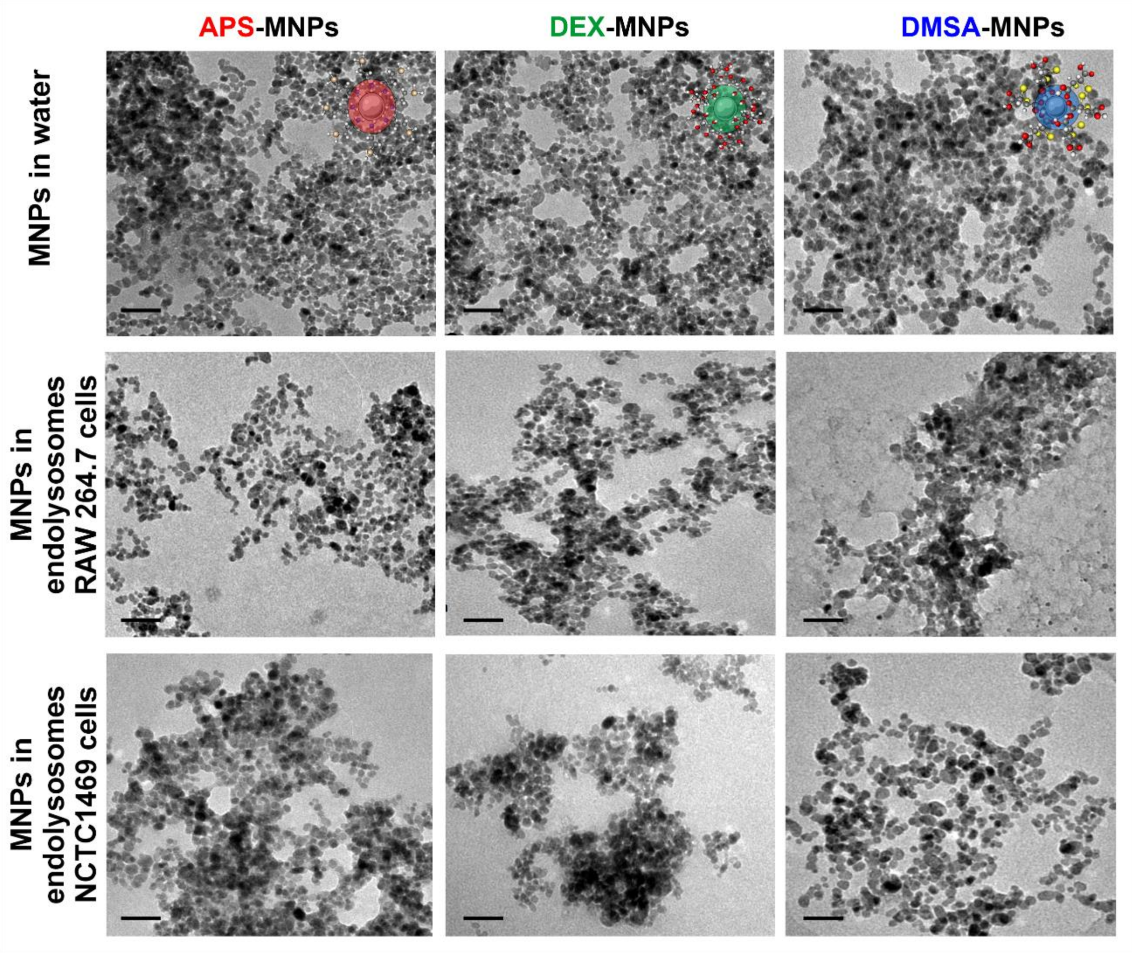

Fig. S7. TEM images of MNPs before and after their internalization in endolysosomes in RAW 264.7 and NCTC1469 macrophagic cells. These images were analyzed to determine the decrease in the size of the iron oxide core as indicative of intracellular degradation. Scale bar 20 nm.

Uptake of MNPs by RAW 264.7 and NCTC1469 cells

We also characterized the uptake of APS-, DEX- and DMSA-MNPs by macrophage cells. The average amount of MNPs internalized by RAW 264.7 cells after 24 h was 50.7 ± 2.3 pgFe/cell for APS-MNPs, 6.5 ± 0.8 pgFe/cell for DEX-MNPs and 61.4 ± 7.1 pgFe/cell for DMSA-MNPs (**Fig. S8a**). The murine liver-derived macrophage-like NCTC1469 cell line internalized more MNPs over 24 h: DMSA-MNPs, 64.7 ± 2.0 pgFe/cell; APS-MNPs, 21.5 ± 6.6 pgFe/cell; and DEX-MNPs, 9.4 ± 4.5 pgFe/cell (**Fig. S8b**).

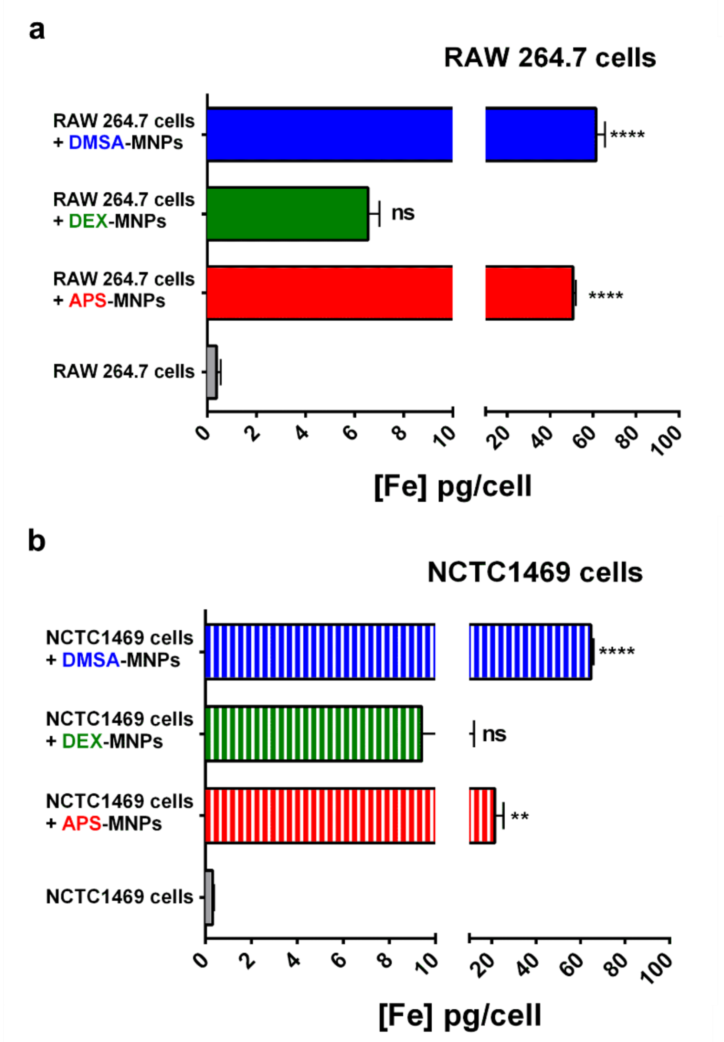

Fig. S8. Cellular iron concentrations in macrophage cells after APS-, DEX- and DMSA-MNP uptake. (a) Quantification of the iron concentration in RAW 264.7 cells by ICP-OES. (b) (a) Quantification of the iron concentration in NCTC1469 cells by ICP-OES. The data are shown as the mean ± SD (n = 3) and the iron concentration in both cell types was compared by a one-way analysis of variance (ANOVA) and Tukey's multiple tests. The asterisks indicate statistically significant differences in iron concentration: ns – no significant differences, *p <0.05, **p <0.01, ***p <0.001, and **** p <0.0001.

Change of the position of the AC magnetic susceptibility maximum as evidence of nanoparticle degradation

The differences in the temperature of the MNP's magnetic susceptibility maximum may be related to a change in the interactions between particles due to less aggregation or to a decrease in the size of particles related to their degradation over time. A larger variation of the maximum temperature location was found in the livers than in the spleens, which may be associated to different particle transformation over time.

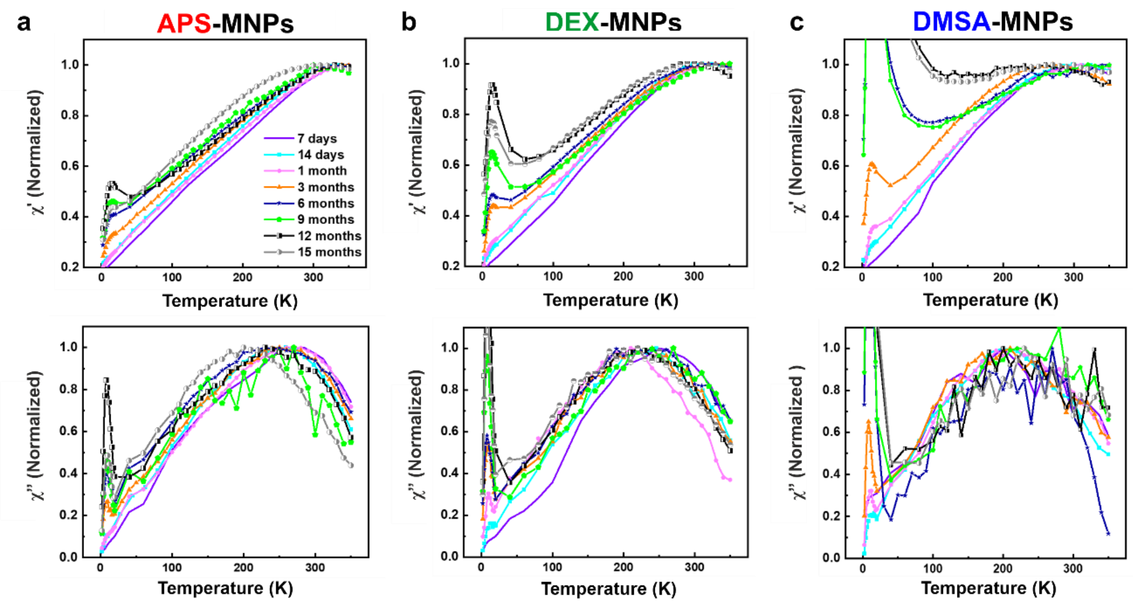

Fig. S9. Temperature dependence of the in-phase and out-of-phase susceptibility scaled to the maximum associated with the presence of particles in the spleen. These plots help visualize the change in position of the AC magnetic susceptibility maximum over time, indicating particle transformation. (a) APS-MNPs, (b) DEX-MNPs and (c) DMSA-MNPs. The in-phase (real, χ' - Top) and out-of-phase (imaginary, χ'' - Bottom) component of the AC magnetic susceptibility measurements are shown.

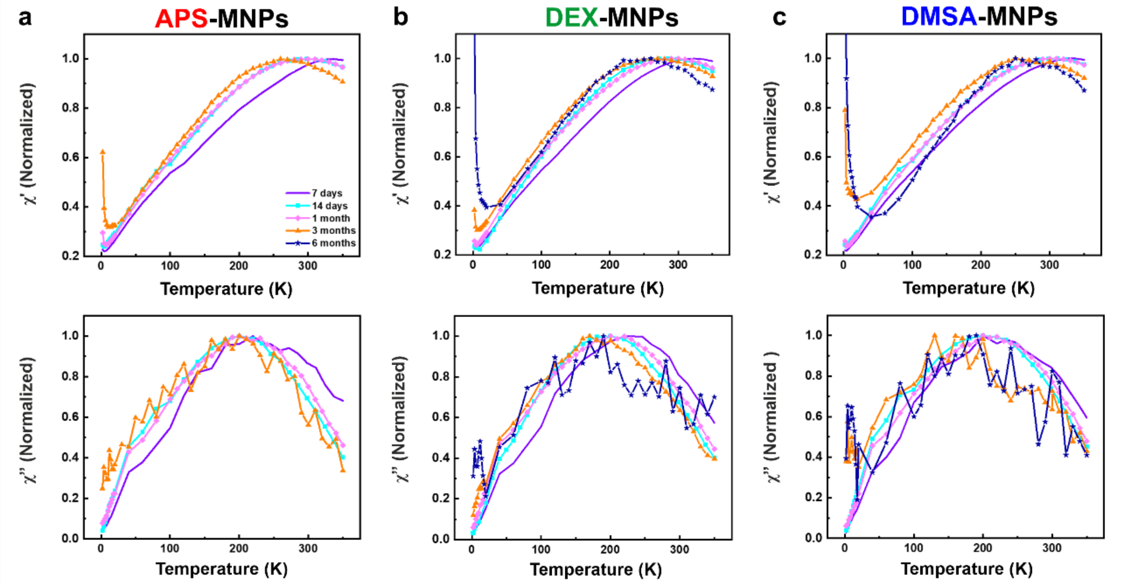

Fig. S10. Temperature dependence of the in-phase and out-of-phase susceptibility scaled to the maximum associated with the presence of particles in the liver. These plots help visualize the change in position of the AC magnetic susceptibility maximum over time indicating particle transformation. (a) APS-MNPs, (b) DEX-MNPs and (c) DMSA-MNPs. The in-phase (real, χ' - Top) and out-of-phase (imaginary, χ'' - Bottom) component of the AC magnetic susceptibility measurements are shown.

Evolution of the ferritin and paramagnetic ion signals in PBS-treated mice at different times of degradation.

The ferritin and paramagnetic ion signal at low temperatures in the liver and spleen of PBS treated-mice was studied at different times by AC magnetic susceptibility (see **Fig. S11**).

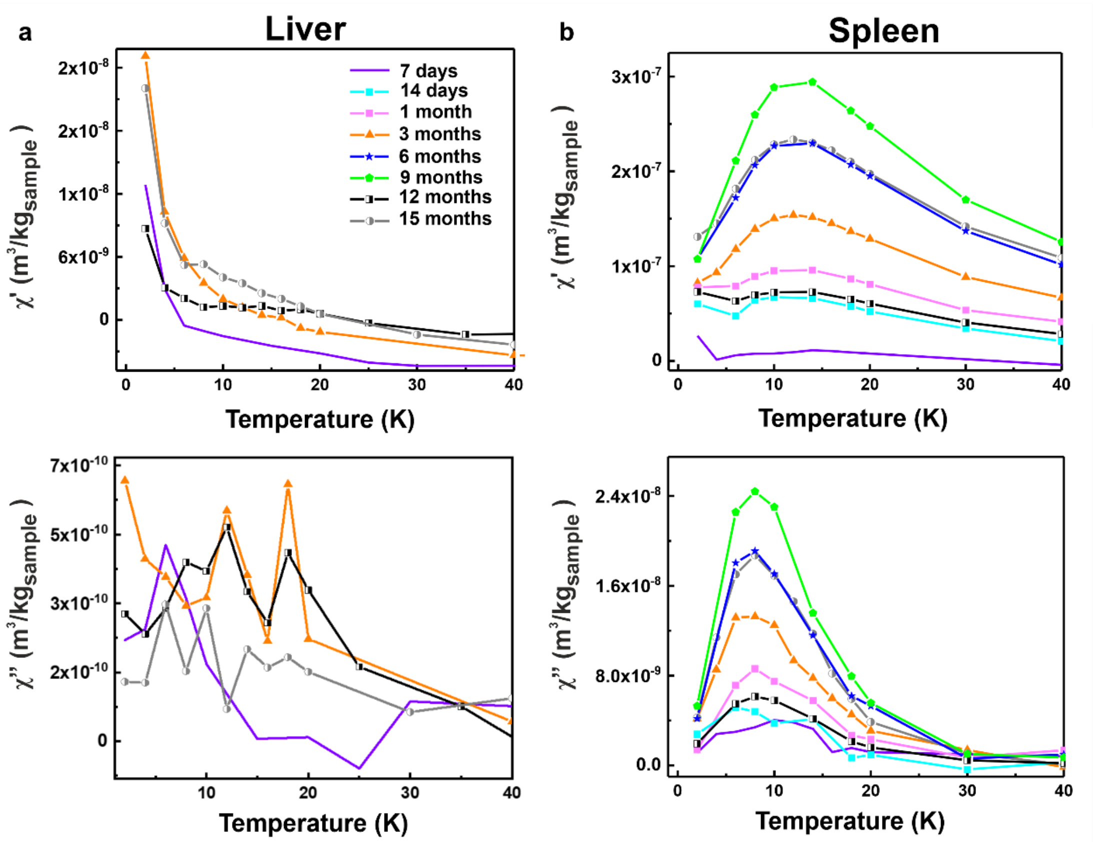

Fig. S11. Evolution of the ferritin and paramagnetic ion signals in PBS-treated mice measured by AC magnetic susceptibility at different times. The magnetic susceptibility showed a paramagnetic signal in the in-phase magnetic susceptibility component in the liver, although no paramagnetic contribution was observed in the spleen. In addition, a ferritin signal in the out-of-phase magnetic susceptibility component that increased over time was observed in the spleen but not in the liver. The in-phase (real, χ' - Top) and out-of-phase (imaginary, χ'' - Bottom) component of the AC magnetic susceptibility measurements of the liver (a) and spleen (b) are shown.

In the liver tissue, a paramagnetic signal in the in-phase magnetic susceptibility component was evident at low temperatures. This signal at low temperature was studied at different times and the ferritin signal was not observed in the out-of-phase magnetic susceptibility in this organ. By contrast, a ferritin signal in the in-phase and out-of-phase magnetic susceptibility components was clearly observed in the spleen, and it increased with time. Nevertheless, no paramagnetic signal was found in the spleen tissue.

Additional file 1. References

1. Portilla Y, Mellid S, Paradela A, Ramos-Fernández A, Daviu N, Sanz-Ortega L, et al. Iron Oxide Nanoparticle Coatings Dictate Cell Outcomes Despite the Influence of Protein Coronas. ACS Applied Materials & Interfaces. 2021;13(7):7924-44.
2. Portilla Y, Mulens-Arias V, Paradela A, Ramos-Fernández A, Pérez-Yagüe S, Morales MP, et al. The surface coating of iron oxide nanoparticles drives their intracellular trafficking and degradation in endolysosomes differently depending on the cell type. Biomaterials. 2022;281:121365.
